# The redox chemistry of La_0.5_Sr_0.5_Cr_0.2_Mn_0.8_O_3−*δ*_ and its application in high capacity anodes of oxygen ion batteries

**DOI:** 10.1039/d6ta00585c

**Published:** 2026-05-20

**Authors:** Barbara Wagner, Alexander Schmid, Stanislaus Breitwieser, Andreas Nenning, Jürgen Fleig

**Affiliations:** a Institute of Chemical Technologies and Analytics, TU Wien Getreidemarkt 9 1060 Wien Austria barbara.wagner@tuwien.ac.at

## Abstract

Solid-state oxygen ion batteries (OIBs) are a novel technology for electrochemical energy storage, based on the exchange of oxygen between two mixed conducting oxide electrodes *via* an oxide ion-conducting electrolyte. Suitable electrode materials not only require good ionic and electronic conductivity, but also a highly variable oxygen non-stoichiometry *δ* to chemically store large amounts of charge. Another desirable characteristic for anodes is good material stability down to very reducing oxygen chemical potentials. This work focuses on the exploration of La_0.5_Sr_0.5_Cr_0.2_Mn_0.8_O_3–*δ*_ and its electrochemical and defect chemical properties, with particular focus on its applicability in anodes of oxygen ion batteries. Thin film model cells were prepared by pulsed laser deposition (PLD) of electrodes on 100-oriented Y:ZrO_2_ single crystals. These planar half-cells were sealed with ZrO_2_ and glass to inhibit oxygen exchange with the atmosphere. Electrode capacities of up to 930 mAh cm^−3^ were achieved and confirmed to be stable over more than 70 cycles at 400 °C between −0.07 V and −2.07 V *vs.* 1 bar O_2_. Charge/discharge curves revealed the existence of two plateaus at −0.8 V and −1.4 V. Further, electrochemical impedance measurements on samples with microelectrodes were employed to study the chemical capacitance *C*_chem_, oxygen diffusion coefficient, and ionic resistivity of La_0.5_Sr_0.5_Cr_0.2_Mn_0.8_O_3–*δ*_ over the same range of potentials. High resolution *C*_chem_*vs.* oxygen chemical potential measurements revealed two clearly separated peaks, indicating two separate redox processes, which correspond to the two distinct plateaus found in the charge/discharge curve. A defect chemical model (Brouwer diagram) was developed, based on a two stage transition: Mn^4+^ → Mn^3+^ → Mn^2+^. The model can quantitatively explain the location of both peaks in the chemical capacitance curve and the corresponding plateaus of the charge/discharge curve. Furthermore, X-ray photoelectron spectroscopic measurements of the Mn^3+^ → Mn^2+^ transition fully confirmed this model. Altogether, this study showed that La_0.5_Sr_0.5_Cr_0.2_Mn_0.8_O_3−*δ*_ is a highly promising anode material for oxygen ion batteries operating at high voltages.

## Abstract 2

Solid-state oxygen ion batteries (OIBs) are a novel technology for electrochemical energy storage, based on the exchange of oxygen between two mixed-conducting oxide electrodes *via* an oxide ion-conducting electrolyte.

## Graphical abstract

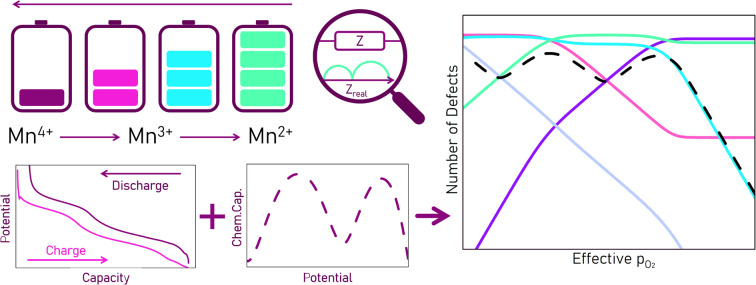

## 1 Introduction

Mixed ionic and electronic conductors (MIECs) with oxygen deficiency play a key role in several energy applications such as high-temperature solid oxide fuel cells (SOFCs), solid oxide electrolysis cells (SOECs), and oxygen permeation membranes. Their ability to transport both oxygen ions and electronic charge carriers is essential for their high-temperature electrochemical functionality. Perovskite oxides, characterized by the general formula ABO_3−*δ*_, are widely studied for these electrochemical energy applications due to their ability to accommodate a variety of point defects and their tunable electronic and ionic conductivity. In oxidic perovskites used in solid state electrochemistry, the A-site is typically occupied by large cations such as lanthanum (La), strontium (Sr) or barium (Ba), while the B-site hosts smaller transition metal cations like manganese (Mn), cobalt (Co), iron (Fe) or chromium (Cr). The interrelations between point defects and chemical potentials in these materials strongly influence their electronic, ionic, and catalytic properties. Accordingly, many studies have focused on investigating the defect chemistry of mixed conducting perovskite materials such as LSM (La_1−*x*_Sr_*x*_MnO_3−*δ*_),^1–4^ LSC (La_1−*x*_Sr_*x*_CoO_3−*δ*_),^5–7^ LSF (La_1−*x*_Sr_*x*_FeO_3−*δ*_),^8–11^ or BSCF (Ba_1−*x*_Sr_*x*_Co_1−*y*_Fe_*y*_O_3−*δ*_)^12–14^ and its consequences regarding their usage in energy applications. When introducing aliovalent cations into the perovskite lattice, charge compensation mechanisms occur to maintain electroneutrality. This compensation mostly occurs through the formation of oxygen vacancies 
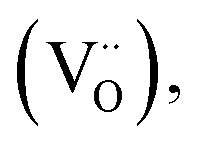
 electronic defects such as electrons (e′) or holes (h˙), or cation vacancies.^15^ In LaFeO_3_, for example, substituting La^3+^ by Sr^2+^ introduces holes, which leads to enhanced p-type conductivity under oxidizing conditions. Under reducing conditions, *i.e.* in low oxygen partial pressures, charge compensation is instead achieved primarily *via* the formation of oxygen vacancies. The predominant compensation mechanism thus depends on the oxygen chemical potential µ_O_ and the resulting variability of the oxygen non-stoichiometry makes it possible to use LSF and other perovskite oxide as electrode materials in solid-state oxygen ion batteries (OIB).^16,17^

Such oxygen ion batteries are based on the exchange of oxygen between two mixed conducting oxide electrodes *via* an oxide ion-conducting electrolyte. Therefore, suitable electrode materials have to exhibit both high ionic and electronic conductivity, as well as a highly variable oxygen non-stoichiometry (*δ*), enabling electrochemical storage of large amounts of charge at typical operation temperatures of 250–500 °C. This charge storage is induced by an externally applied voltage, rather than variations in the oxygen partial pressure. The capacity for charge storage is determined by the defect chemistry of the material, particularly the concentration of oxygen vacancies, electrons, and holes. Therefore, a crucial step towards the development of functional OIB cells is the understanding of the defect chemical reactions in potential electrode materials across a wide oxygen chemical potential range and thus a wide voltage range.

Anode materials are expected to exhibit high oxygen vacancy concentration changes under very reducing conditions, preferably at voltages even more negative than −1 V *vs.* 1 bar O_2_. Lanthanum strontium manganite (LSM, La_1−*x*_Sr_*x*_MnO_3_) and lanthanum strontium chromite (LSCr, La_1−*x*_Sr_*x*_CrO_3_) are two prominent examples of doped perovskites used in energy technologies, particularly in solid oxide fuel cells (SOFCs). LSM often functions effectively as a cathode due to its high electronic conductivity and catalytic activity for oxygen reduction,^18–21^ while LSCr is explored as an interconnect materials for its stability in both oxidizing and reducing atmospheres.^20^ However, their applicability in next-generation devices such as oxygen ion batteries (OIBs) demands a reassessment of their redox stability and conductivity at very low oxygen chemical potentials.

Looking at La_1−*x*_Sr_*x*_CrO_3_, although it is stable in reducing atmospheres, it has a rather low electronic conductivity, making it less suitable for OIBs.^22^ La_1−*x*_Sr_*x*_MnO_3−*δ*_ (LSM) on the other hand, performs robustly in typical SOFC cathode conditions, but decomposes at voltages significantly below −1 V *vs.* O_2_, which are desired for oxygen ion battery anodes. In a detailed study by Mizusaki *et al.*,^1^ the oxygen non-stoichiometry and decomposition characteristics of La_0.6_Sr_0.4_MnO_3−*δ*_ were probed through coulometric titration and thermogravimetry, revealing two stable *δ* plateaus at 0 and approximately 0.0225. Upon further reduction, LSM decomposes at *δ* < 2.8 into (La_0.6_Sr_0.4_)_2_MnO_4_ and MnO, with an inferred oxygen content of ∼2.5 per Mn atom (*δ* ≈ 0.5). Extrapolation of this data to lower temperatures yields a decomposition oxygen partial pressure of *ca.* 10^−34^ bar at 400 °C (673 K), equivalent to an electrochemical potential of −1.12 V *vs.* 1 bar O_2_, as calculated from Nernst's equation. This is insufficient for oxygen ion battery anodes, which should operate at voltages much below −1 V *vs.* O_2_ in air. These shortcomings motivate the search for alternative compositions that can maintain structural integrity under even lower oxygen chemical potentials. Various doping strategies may be applied, such as Cr-doping of LSM to synthesize La_1−*x*_Sr_*x*_Cr_*y*_Mn_1−*y*_O_3−*δ*_, where the incorporation of Cr^3+^ is intended to stabilize reduced Mn^2+^ ions in the perovskite lattice and prevent phase decomposition.^23,24^ In our preliminary studies, LSCrMn (La_0.5_Sr_0.5_Cr_0.2_Mn_0.8_O_3−*δ*_) has been identified as a particularly promising candidate for OIB anodes, which may still work at chemical potentials where LSM would fail.

In this study, we use complementary electrochemical impedance and DC measurements on LSCrMn thin film model electrodes with blocked surface oxygen exchange kinetics (planar Pt/Ti or ZrO_2_/glass) to reveal its charge/discharge behaviour in oxygen ion batteries and elucidate the underlying defect chemistry. We show that LSCrMn exhibits a surprisingly wide reversible redox window down to −2.07 V *vs.* 1 bar O_2_ at 400 °C, which is stable over more than 70 redox cycles. A full defect chemical model (Brouwer diagram) is presented, which explains this high electrode capacity as a result of a two stage transition of Mn^4+^ → Mn^3+^ → Mn^2+^. Additional X-ray photoelectron spectroscopy (XPS) measurements of the Mn^3+^ → Mn^2+^ transition further validate this model.

## 2 Results and discussion

### 2.1 Chemical and structural characterization

**Fig. 1:**
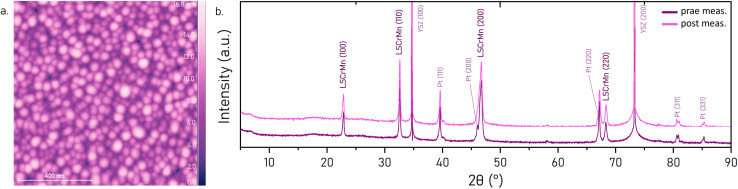
(a) Atomic force microscopy (AFM) image of the LSCrMn thin film surface, (b) prae and post cycling Bragg–Brentano XRD patterns of an LSCrMn thin films with a Pt current collector on top on YSZ (100), showing polycrystalline growth of the film and no structural post mortem changes.

Bragg–Brentano X-ray diffraction (XRD) measurements were performed on prae and post measurement La_0.5_Sr_0.5_Cr_0.2_Mn_0.8_O_3−*δ*_ thin films deposited onto an yttria stabilized zirconia (YSZ) single-crystal substrate with a Pt current collector on top (see Fig. 1b) in a *θ*–2*θ* range of 5–90°. The results indicate that the perovskite-type LSCrMn film is polycrystalline. The post-cycling XRD pattern shows no additional reflections and no evidence of secondary phase formation, confirming the preservation of the perovskite structure upon repeated reduction and oxidation. Only minor peak shifts were observed, ranging from 0.01° for the (100) reflection at 22.87° (22.86° post mortem) to 0.08° for the (220) reflection at 68.35° (68.28° post mortem). These shifts are small and fully reversible within experimental uncertainty, and are therefore attributed to reversible lattice expansion/contraction associated with changes in oxygen non-stoichiometry rather than irreversible lattice distortion. Inductively coupled plasma mass spectrometry (ICP-MS) confirmed that the composition of the film is in close agreement with the target stoichiometry. Fig. 1a shows an image of the surface characterization by atomic force microscopy, revealing a granular morphology, indicative of polycrystalline, columnar growth with relatively small grain sizes between 28 and 57 nm. Such a morphology is also found for closely related dense perovskite films grown by PLD.^25,26^

### 2.2 DC charge/discharge curves

To assess the redox cycling behavior, a LSCrMn thin film electrode on a half-cell sample (see Experimental Sample Preparation and Analysis) was galvanostatically cycled with a constant current of 37 µA cm^−2^ for 71 cycles.

**Fig. 2:**
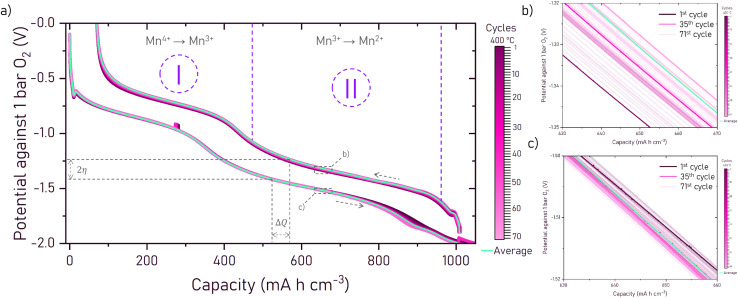
(a) Galvanostatic charge/discharge profiles of La_0.5_Sr_0.5_Cr_0.2_Mn_0.8_O_3−*δ*_ thin-film electrodes on YSZ, measured at 400 °C over 71 consecutive half-cell cycles (charge: 1013 mAh cm^−3^, discharge: 938 mAh cm^−3^). The overlapping purple-to-pink curves show individual cycles, while the turquoise line indicates the averaged voltage profile. Two distinct discharge plateaus labeled I and II reflect the stepwise reduction of Mn^4+^ to Mn^3+^ and Mn^2+^. The marked plateaus are only labeled for the discharge curve for simplicity purposes. Voltages are measured between WE and CE/RE and recalculated to 1 bar O_2_. The overpotential *η* is determined from the difference between charge and discharge curve with the charge offset Δ*Q* considered as shown. (b) Close up of the discharge curve from −1.32 to −1.35 V/630–670 mAh cm^−3^, with the 1st, 35th, 71st and average cycle marked bold. (c) Close up of the charge curve from −1.5 to −1.52 V/630–660 mAh cm^−3^, with the 1st, 35th, 71st and average cycle marked bold.

Fig. 2 shows the measured DC half-cell charge/discharge curves from −0.07 to −2.07 V *vs.* 1 bar O_2_ of a La_0.5_Sr_0.5_Cr_0.2_Mn_0.8_O_3−*δ*_ thin film electrode on a YSZ single-crystal electrolyte at 400 °C. A total of 71 charge and discharge cycles are plotted as overlapping purple-to-pink curves, showing the very good cycling stability of the half cell. The turquoise curve shows the averaged voltage profile. The total capacity of the charge curve remains constant over all cycles. All given potentials reflect voltages between working and reference electrode normalized to 1 bar O_2_*via* Nernst's equation, *i.e.* shifting the measured voltage (in *p*_O_2_,chamber_ = 10 mbar) by *U*_CE,shift_ = −0.07 V:1
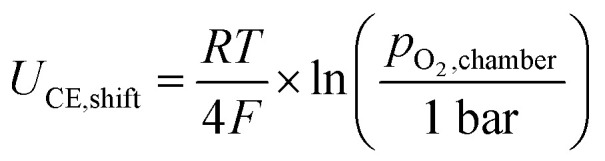


The charge curve exhibits a distinct initial drop to a potential of around −0.70 V and reaches Plateau I at −0.81 V, determined by the shallow slope inflection point of the curve. The slope increases again before the curve reaches its first inflection point at 354 mAh cm^−3^ and −1.12 V. Plateau II is observed at −1.49 V and ends with the second inflection point of the curve at 887 mAh cm^−3^. This second inflection point, which indicates a third plateau at very reducing conditions, does not appear at first and becomes more pronounced with each cycle. The nature of it is not yet known. However, as it can be seen in Fig. 2, it does not affect the total capacity reached during charging. This total capacity remains almost constant over all cycles and averages to a total of 1013 mAh cm^−3^. The overall maximum reduction potential of −2.07 V is limited by the electrolytic window of the YSZ electrolyte.

The total capacity of the discharge curve remains almost constant over all cycles as well, reaching an average of 938 mAh cm^−3^. The discharge curve exhibits a small initial rise to a potential of −1.72 V, similar to the initial decrease in the charge curve, though less prominent. The rest of the discharge curve resembles the charge curve in shape and size with the plateaus lying at I: −0.71 V and II: −1.39 V. No indication of a third plateau is visible. Table 1 summarize the voltages of the two plateaus and Table 2 the voltages of the inflection points/limits of the two identified regions and also compares them with plateaus identified from chemical capacitance measurements (*C*_chem_), which are discussed later in more detail.

**Table 1:** Summary of the positions of the two plateaus observed in the measured battery curve. The charge and discharge values include the contribution from the overpotential, while the *C*_chem_ value corresponds to the maxima of the respective voltage dependencies

	Plateau I	Plateau II
Discharge	−0.71 V	−1.39 V
Charge	−0.81 V	−1.49 V
*C*_chem_	−0.79 V	−1.45 V

**Table 2:** The limits of the two observed plateaus in the measured battery curve. The charge and discharge values include the contribution from the overpotential, while the *C*_chem_ value corresponds to the minima of the respective voltage dependencies. Limit I refers to the end of the region in which the Mn^4+^ → Mn^3+^ redox reaction occurs and Limit II to the Mn^3+^ → Mn^2+^ redox reaction

	Limit I	Limit II
Discharge	−1.01 V	−1.72 V
Charge	−1.15 V	−1.84 V
*C*_chem_	−1.07 V	−1.86 V

The charge/discharge curves in full exhibit a noticeable asymmetry, which does not change during cycling. More specifically, the charging capacity slightly exceeds the discharge capacity by Δ*Q* = 81 mAh cm^−3^, resulting in an average coulomb efficiency of 92% over 71 cycles. In contrast to this only moderate Faraday efficiency, the cycling stability is good. This suggests that a kind of leakage process (electron conduction in YSZ or imperfect blocking of O_2_ incorporation through the glass sealing under very reducing conditions) rather than a decomposition reaction causes the deviation from perfect Faraday efficiency. To further validate this, post mortem XRD measurements of a cycled sample have been performed (see Fig. 1b). The absence of new diffraction peaks and the presence of only minor, reversible peak shifts confirm that little to no irreversible structural degradation occurred during cycling. Since this irreversible charge seems to flow mainly during the charge cycle, we consider the discharge curve for defect chemical interpretations in the following. When analyzing only the discharge capacities, rather than comparing charge and discharge capacities directly, we also see that irreversible capacity losses due to material degradation are below 1% during 71 cycles, (1st cycle = 941.35 mAh cm^−3^, 71st cycle = 936.17 mAh cm^−3^) indicating reversible electrochemical behavior of the electrode material itself. Please note that earlier measurements down to only −1.1 V *vs.* 1 bar O_2_ showed Faraday efficiencies close to 100%.^17^

Moreover, an overpotential *η* of about ± 0.09 V can be deduced from the charging and discharging curves, originating mostly at the thick YSZ electrolyte. Please note that for determining *η* the offset of the charge/discharge curve Δ*Q* has to be considered as well (see Fig. 2).

The discharge curve also exhibits two well separated charge/discharge plateaus labeled I and II, which have an average range of 377 mAh cm^−3^ and 561 mAh cm^−3^, respectively. These values correspond to charge transfers of approximately 0.54 (Plateau I) and 0.81 (Plateau II) electrons per unit cell (e^−^/u.c.), respectively (see Table 3). As we discuss in more detail below (see Electrochemical Impedance Measurements), these inflection points correspond to the minima of the chemical capacitance which are at open circuit voltages of −1.07 V and −1.82 V *vs.* O_2_.

**Table 3:** Summary of the electrons per unit cell, determined for each region of the DC measured and from the EIS reconstructed battery curves, compared with the theoretical values calculated from the chemical equations

	e^−^/u.c. measured	e^−^/u.c. from equation
DC-plateau I	0.54	0.5
EIS-plateau I	0.56	
DC-plateau II	0.81	0.8
EIS-plateau II	0.78	

The capacity values of the two plateaus show a striking correspondence to the electrode composition: the first plateau – 0.54 e^−^/u.c. – is very similar to the Sr dopant concentration of 0.5. As described above (Introduction), at high to moderate oxygen chemical potentials the acceptor doping is compensated by the introduction of electron holes, *i.e.* by partial oxidation of a B site cation (likely Mn). In our case, this results in 0.5 Mn^4+^/u.c., while the remaining 0.3 Mn/u.c. are in the 3+ oxidation state. Thus, the transition from hole to vacancy compensation, *i.e.* the reduction of all Mn^4+^ → Mn^3+^ requires 0.5 e^−^/u.c, which excellently matches the measured charge of Plateau I. Thus, we assume that Plateau I primarily refers to the Mn^4+^ → Mn^3+^ redox reaction.

The second plateau – 0.81 e^−^/u.c. – corresponds almost perfectly to the total manganese content of the electrode of 0.8 Mn/u.c. Therefore, we conclude that it reflects a further reduction step of all Mn ions from Mn^3+^ → Mn^2+^. We may thus formulate for plateaus I and II:2
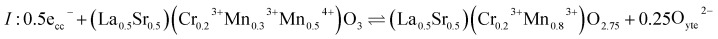
3
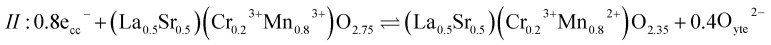
with subscript cc and yte representing the current collector and the electrolyte. The excellent agreement between the measured capacities and the theoretical number of electrons per unit cell supports the assumption that the reversible redox activity of Mn is the predominant contributor to charge storage, while Cr remains redox inactive. This is also in agreement with previous experimental X-ray absorption spectroscopy results of LSCrMn with different Sr contents. These showed that compensation of Sr and oxygen vacancy formation is associated only by changes of oxidation states of Mn ions, while the Cr ions stay in their trivalent oxidation state unchanged.^27^ The high stability and reversibility of the charge discharge curves implies that the perovskite lattice tolerates these Mn oxidation state changes and the very large oxygen non-stoichiometry *δ* of 0.65, without undergoing any structural degradation.

Accordingly, even though Cr remains in the 3+ state over the entire voltage range, its presence is still required for its stabilizing effect. Without any chromium content, *i.e.* in the case of LSM, the material is far less stable with respect to reduction and undergoes phase decomposition at potentials around −1.12 V *vs.* 1 bar O_2_ (equivalent to a *p*_O_2__ of 10^−34^ bar) at 400 °C (see Introduction). Up to this decomposition potential, manganese only gets reduced to Mn^3+^.^1^ With the additional chromium doping the perovskite structure of LSCrMn is stable down to potentials of −2.07 V against 1 bar O_2_, *i.e.* 10^−57^ bar *p*_O_2__ at 400 °C, and Mn can be further reduced to Mn^2+^. Altogether we thus have an anode material with two plateaus (−0.8 V and −1.4 V), a remarkably high capacity and more than 60% of this capacity being in the −1.4 V plateau with an upper voltage limit around −1.8 V.

### 2.3 Electrochemical impedance measurements

**Fig. 3:**
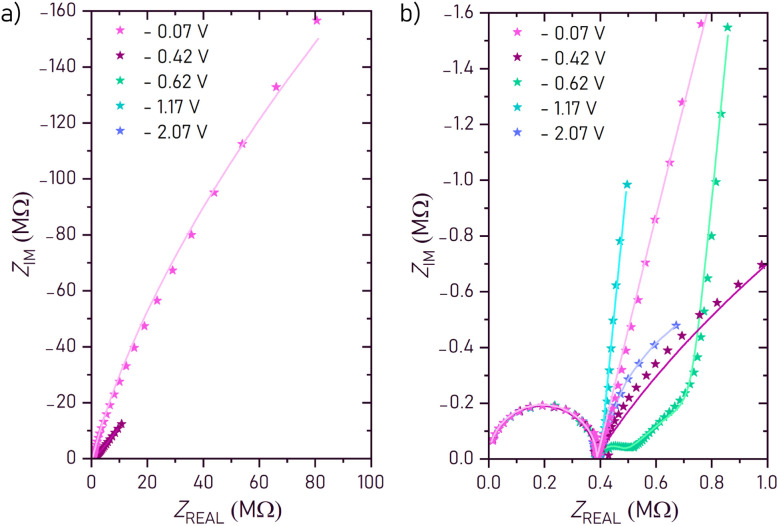
Impedance spectra measured at 400 °C for different applied voltages, and their corresponding fits, using the modified Randles' equivalent circuit: (a) impedance spectra at various voltages. (b) Magnification with particular focus on the intermediate frequency semicircle.

**Fig. 4:**
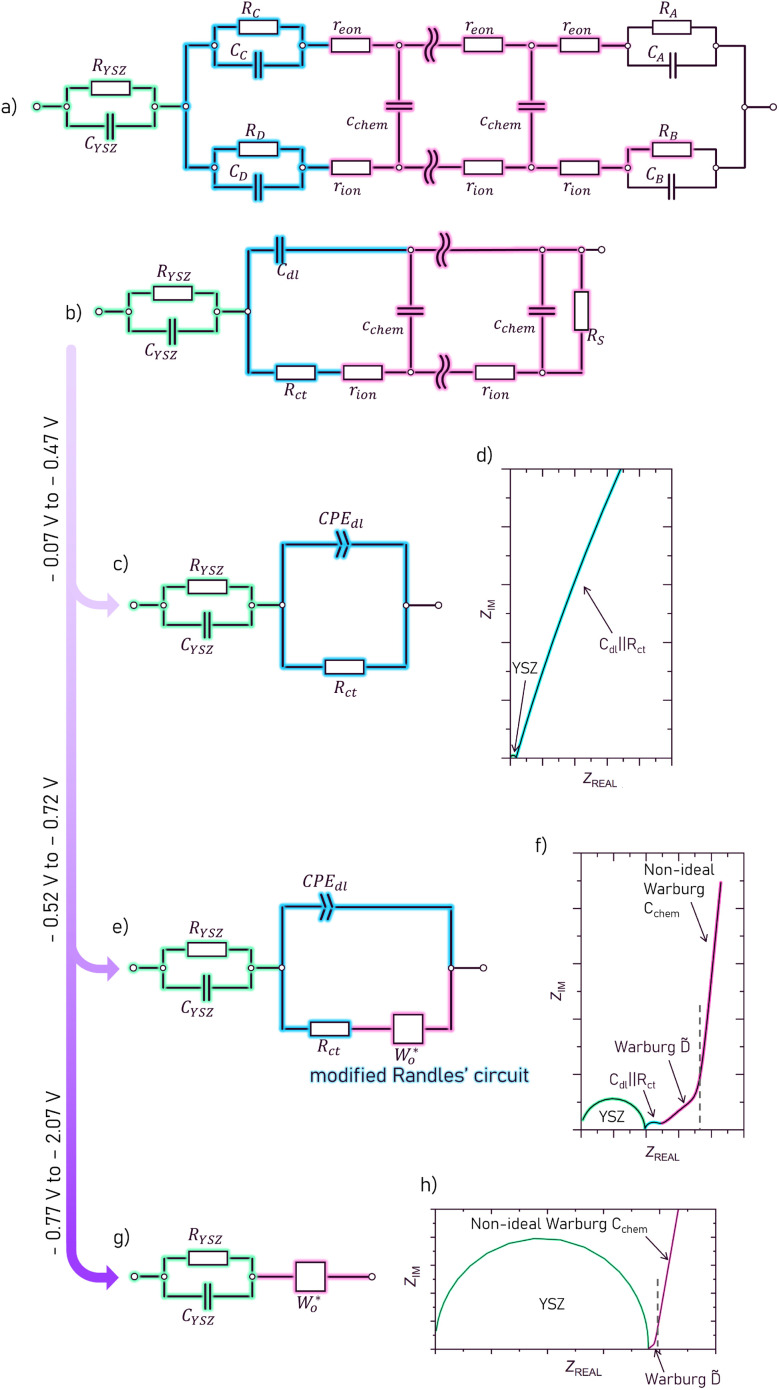
(a) A general one-dimensional transmission line of a mixed conductor on top of an electron blocking electrolyte and an ion blocking current collector. (b) First simplification step from the general transmission line (c) Further simplified transmission line (from b)) with an R ‖ C and an R ‖ CPE element remaining, used for the voltage range from −0.07 V to −0.47 V (d) Example impedance curve between −0.07 V to −0.47 V with all transmission line elements marked in their respective colors (e) Further simplified transmission line (from b)) – Modified Randles' circuit with an open non-ideal Warburg element for the path marked in pink (f) example impedance curve between −0.52 to −0.72 V with all transmission line elements marked in their respective colors (g) further simplified transmission line (from b)) with an R ‖ C and a non-ideal open Warburg element remaining, used for the voltage range below −0.77 V. (h) Example impedance curve below −0.77 V with all transmission line elements marked in their respective colors.

For a better insight into the oxygen storage kinetics of LSCrMn, bias dependent impedance spectra were acquired on a LSCrMn micro-electrode sample. Fig. 3a and b show exemplary impedance spectra for different potentials in which several frequency features can be identified. All potentials reflect voltages between working electrode (WE) and counter electrode (CE), normalized to 1 bar O_2_. The high-frequency semicircle stays constant over the whole voltage range and can be assigned to the YSZ electrolyte. It is therefore considered in all used equivalent circuits (see Fig. 4) by a R_YSZ_‖CPE_YSZ_ element (CPE = constant phase element). The extracted conductivity *σ* and permittivity *ε* values of the YSZ layer are in good agreement with values reported in literature, which supports the assignment of this feature.^28,29^

The shape and size of the low-frequency feature varies drastically with bias voltage, and these changes reflect µ_O_ dependent oxygen storage and conduction kinetics in the electrode. The size of the respective impedance features varies drastically with bias, and separation of them is only possible in a limited potential range. The separation works best for the spectrum at −0.62 V (green), where three features are distinguishable: an intermediate-frequency arc, a 45° line, and an almost vertical capacitive feature at lowest frequencies. The intermediate-frequency arc is attributed to the charge transfer resistance and the interfacial capacitance at the electrode–electrolyte interface. It dominates the low-frequency response between −0.07 V and −0.47 V, but shrinks drastically with more negative voltage, disappearing completely below −0.72 V. As they are not the main focus of this work, but valuable and interesting data nonetheless, the charge transfer resistance and interfacial capacity are discussed in more detail in the SI 1. In the limited potential range where it is visible, it is considered in the equivalent circuit as *R*_ct_, the charge transfer resistance, and CPE_int_ (constant phase element), reflecting the interfacial capacitance at the electrode–electrolyte interface. At −0.52,V a 45° slope and bend become visible and are attributed to a non-ideal open Warburg element 
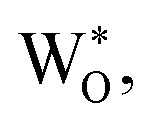
 which we assign to the ambipolar oxygen diffusion through the electrode material. The full 
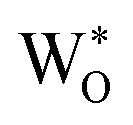
 feature consists of the slope and the nearly vertical rise afterwards attributed to the chemical capacitance *C*_chem_ of the mixed conducting working electrode.

For a mechanistic interpretation and understanding of the deduced fit parameters, we correlate these fit circuits to the general transmission line model of mixed conductors (Fig. 4a) by applying appropriate boundary conditions and simplifications.^30^ This transmission line considers the mixed conductivity by differential ionic (*r*_ion_) and electronic (*r*_eon_) resistances and also includes chemical capacitances *C*_chem_, describing the ability for stoichiometric changes. We assume that the electronic conductivity (*σ*_eon_) of the MIEC is much higher than the ionic conductivity (*σ*_ion_). The electronic resistances *r*_eon_ in the transmission line can therefore be neglected and the rail is replaced by a short circuit. On top of the mixed conductor thin film, the Pt/Ti current collector functions as an ion-blocking boundary (*R*_B_ → ∞, *C*_B_ → 0) which is reversibly transmissive for electrons (*R*_A_ → 0, *C*_A_ → ∞). The electrode–electrolyte interface, marked in blue in the transmission line, on the other hand, is electron-blocking and has an interfacial double layer capacitance (*C*_D_), which gives rise to *R*_C_ → ∞ and *C*_C_ → *C*_int_. A transport of ions is possible *via* the corresponding charge transfer resistance (*R*_D_ → *R*_ct_). The other interface capacitance (*C*_D_) is neglected in comparison to the interface capacitance *C*_int_, (*C*_D_ → 0). The electrolyte can be considered by a *R*_YSZ_‖C_YSZ_ element in series to the transmission line, given that its relaxation frequency is much higher than that of the electrode.

The resulting circuit (Fig. 4b) can be further simplified to three cases:

1. A circuit with serial R ‖ C and R ‖ CPE elements (Fig. 4c), when considering the voltage range from −0.07 V to −0.47 V in which the charge transfer resistance and interfacial capacitance (*C*_int_) are the dominant features.

2. A so called modified Randles' circuit (Fig. 4e), which combines a non-ideal open Warburg element serial to the charge transfer resistance, with the interfacial capacitance parallel to both of these elements. This circuit considers the voltage range from −0.52 V to −0.72 V, when both the intermediate frequency semi-circle and the low frequency Warburg feature are visible in the spectra.

3. And an R ‖ C element in series with just the non-ideal open Warburg element, which considers the spectra case below −0.72 V, when only the high frequency YSZ and the Warburg feature remain (Fig. 4g).

Since neither the chemical capacitance nor the diffusion coefficient or ionic resistivity can be extracted in case (1) the following explanation focuses on case (2) and (3), in particular on the non-ideal open Warburg element 
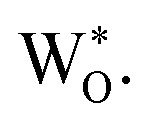
 It utilizes a so called modified Randles' circuit (Fig. 4g), by combining the differential ionic resistance and differential constant phase elements into an open Warburg element with impedance:4
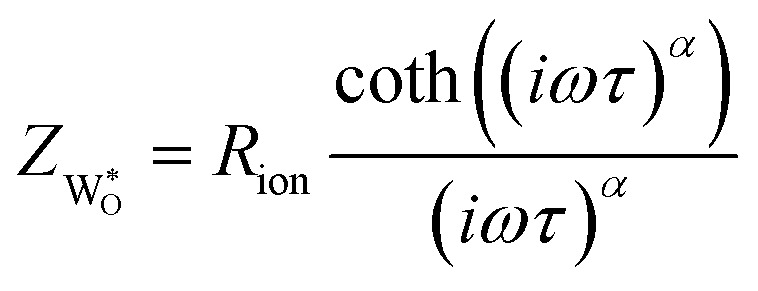
which describes the one-dimensional finite-space diffusion, as derived from Fick's laws.^31^ The impedance of the Warburg element process consists of an ionic resistance *R*_ion_, a specific time constant *τ* and a non-ideality factor *α*, which is *α* = 0.5 for an ideal Warburg impedance. Exact reasons behind this non-ideality of 
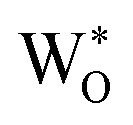
 are not known, possibly it is partly caused by a non-ideal blocking of ions at the surface (*R*_B_ = *R*_S_ ≠ ∞) (*i.e.* an imperfect sealing by the Pt/Ti top layer).

The chemical or ambipolar diffusion coefficient *D̃* can be calculated from the inverse time constant and the characteristic length of the material, *i.e.* the film thickness *L via*:^32^5
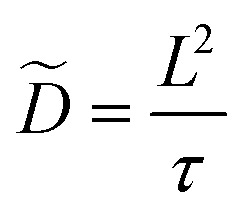


The time constant *τ* is also the product of the ionic resistance and the chemical capacitance of the mixed conducting electrode:

6 τ = *R*_ion_*C*_chem_

From the fit parameters of 
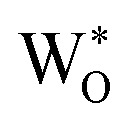
 (*τ* and *R*_ion_) we can thus also calculate *C*_chem_. The chemical capacitance of a MIEC oxide, *C*_chem_, is a key parameter that characterizes its ability to store charge through changes in its chemical composition *i.e.* the concentration of charge carriers (such as ions or electrons), in response to a changing oxygen chemical potential µ_O_.^30^

**Fig. 5:**
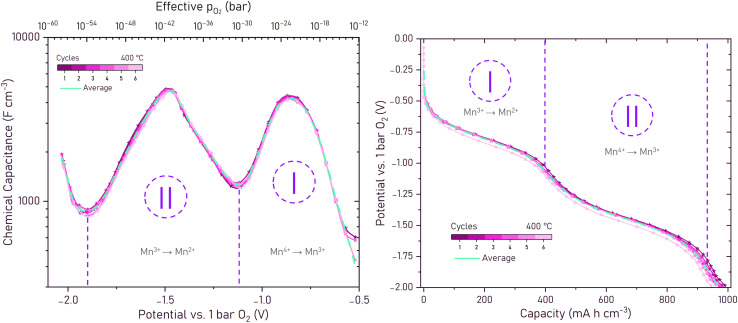
(a) Measured chemical capacitance at 400 °C derived from impedance spectra at various potentials between −0.07 and −2.07 V *vs.* 1 bar O_2_, fitted using the modified Randles' or 2 arc equivalent circuits. The figure displays values of six cycles, highlighting the variations in capacitance with each cycle, as well as the average chemical capacitance across all cycles. The regions I and II with peaks in the capacitance indicate the transitions between different Mn redox states. (b) Reconstructed charge/discharge curves calculated from the chemical capacitance values derived from impedance spectra. Two distinct plateaus arise at −0.79 V and −1.45 V with inflection points at −1.07 V and −1.82 V, limiting the regions I and II.

Fig. 5a and b show the *C*_chem_ fit values of six charge/discharge cycles in total and the average across all cycles. The data from six measurement cycles are displayed in various shades of pink, whereas the averaged values are depicted in teal, allowing for a visual comparison of individual variability and the overall electrochemical trends. The total battery charge was determined by integrating over the chemical capacitance:7
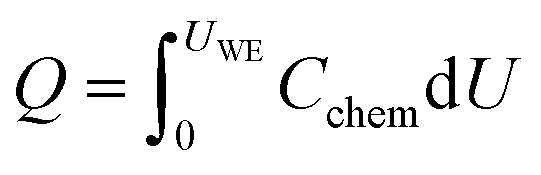


This allowed us to reconstruct the charge/discharge curve of the electrode (Fig. 5b) from the impedance spectra. In Fig. 5a, two distinctive peaks are visible, one exhibiting an average 4415 F cm^−3^ at its highest point at −0.79 V and another one with 4777 F cm^−3^ at −1.45 V. The calculated charge/discharge curve (see Fig. 5b) exhibits plateaus at the same voltage values. These values agree excellently with those obtained by directly measured charge/discharge curves (see Fig. 2 and Table 1). Additionally, two characteristic minima are clearly observable in the data, located at 1250 F cm^−3^ at −1.07 V and 852 F cm^−3^ at −1.86 V, respectively. These minima are the inflection points of the calculated capacity curve and are used to separate the two distinct electrochemical regions labeled I and II in Fig. 5a and b. The values further correspond excellently to the voltage values of the inflection points identified in the directly measured DC charge/discharge curves (see Table 2).

These two regions represent the sequential reduction of manganese within the perovskite lattice under increasingly negative voltages. Region I, spanning the potential range between 0 to −1.07 V, corresponds to the reduction of Mn^4+^ to Mn^3+^, while Region II, between −1.07 V and −1.82 V, represents the subsequent reduction of Mn^3+^ to Mn^2+^. A third increase in *C*_chem_ is visible below −1.86 V. However, its origin is not yet fully understood. It may be attributed either to the formation of trapped electrons or single charged oxygen vacancies 
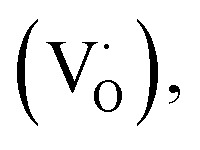
 or to the onset of chromium reduction from Cr^3+^ to Cr^2+^. This feature is, however, fully reversible when cycling the potential between −2.07 and −0.07 V, suggesting that no permanent structural or chemical degradation occurs in this regime. Applied potentials below −2.17 V, on the other hand, cause not only electronic conductivity of the YSZ electrolyte, but also permanent damage by degradation to the LSCrMn thin film and a significant loss in cyclability, chemical capacitance and overall capacity.

The reconstructed charge/discharge curves in Fig. 5b predict an average electrode capacity of 970 mA h cm^−3^ at voltages as low as −2.07 V. Within the boundaries given by the minima of Fig. 5a capacities of 397 (Region I) and 544 (Region II) mA h cm^−3^ are reached. The electrons per unit cell calculated from these capacity values correspond to 0.56 (Region I) and 0.78 (Region II) e^−^/u.c. respectively (see Table 3). These values also match well with the expected values calculated from the chemical equation and the ones from the previously measured DC battery curves.

**Fig. 6:**
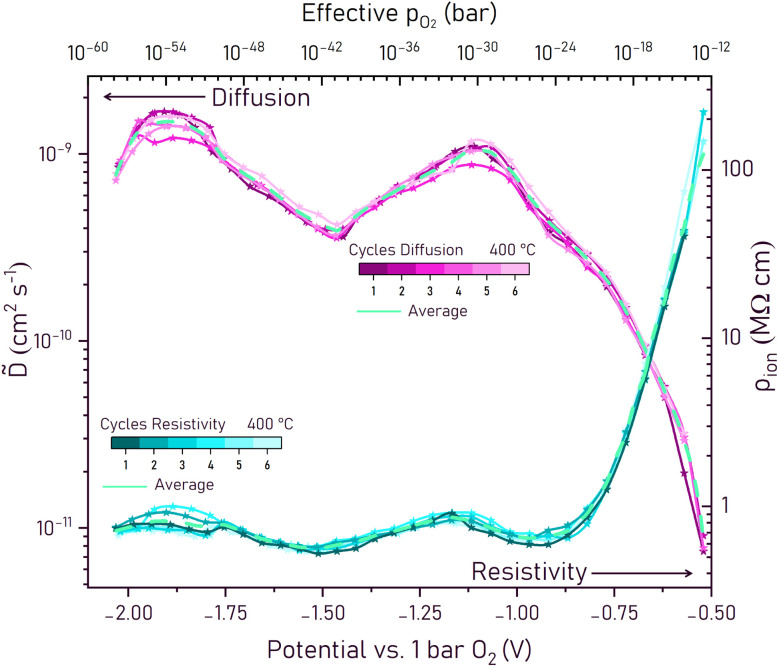
Chemical diffusion coefficient *D̃* and ionic resistivity *ρ*_ion_ at 400 °C calculated from impedance spectra and fits, shown as values across six cycles with the average for each.

The impedance data not only includes the thermodynamic information (*C*_chem_), but also kinetic data on ionic motion and oxygen chemical diffusion. Fig. 6 shows the fit values of the chemical diffusion coefficient *D̃* and the ionic resistivity (*ρ*_ion_) against the potential between −0.52 and −2.07 V. Those are calculated from the fitted time constant *τ* and *R*_ion_ of the 
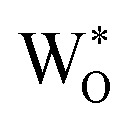
 element, the known active electrode area *A* and the electrode thickness *L* using eqn (5) and (6). All calculated values and the average across all six cycles are plotted in Fig. 6.

The diffusion coefficient could first be fitted at −0.52 V and starts at 7.4 × 10^−12^ cm^2^ s^−1^. Qualitatively, it follows the shape of the chemical capacitance curve inversely, meaning the maxima and minima positions are switched. It increases until −1.07 V, where it reaches a peak of around 10^−9^ cm^2^ s^−1^ and decreases again until −1.45 V to a value of 4 × 10^−10^ cm^2^ s^−1^. The second visible peak is at −1.86 V at 1.5 × 10^−9^ cm^2^ s^−1^. The chemical diffusion itself is proportional to (*R*_ion_*C*_chem_)^−1^ (of eqn (5) and (6)) and for little variation of *R*_ion_ it shows the voltage dependence of *C*_chem_^−1^. However, tracing *D̃* back to defect chemical properties can be non-trivial and an in depth interpretation is beyond the scope of this study.

The resistivity decreases from around 200 MΩ cm at −0.5 V to a broad “plateau” with only little variation around 0.6 MΩ cm at −0.8 V. This resistivity decrease most probably reflects the substantial increase of the oxygen vacancy concentration towards more negative voltages. Indeed, the defect chemical analysis of *C*_chem_, with determination of a Brouwer diagram, suggests a transition to a plateau of the vacancy concentration at −0.8 V (see below). A detailed interpretation of the remaining slight increases and decreases of *ρ*_ion_ below *ca.* −1 V are beyond the scope of this paper. It might be caused by vacancy mobility changes due to varying defect interactions (varying Mn^2+^, Mn^3+^, Mn^4+^, concentrations) and the further increase of the oxygen vacancy concentration in regime II. Please note that this ionic resistivity for high vacancy concentrations is still much worse than that of YSZ (*ca.* 10^4^ Ω cm for 8 mol% Y_2_O_3_ at 400 °C).^33^ Altogether, in the voltage ranges relevant for oxygen ion batteries (below −0.8 V) the resistivity at 400 °C is thus in a range to allow current densities of about 10 mA cm^−2^ at an overpotential of *ca.* 40 mV (calculated for 1/3 of 300 nm electrode thickness).^34^ Better ionic conductivities are certainly desirable to enable thicker electrodes or higher currents.

### 2.4 XPS measurements

**Fig. 7:**
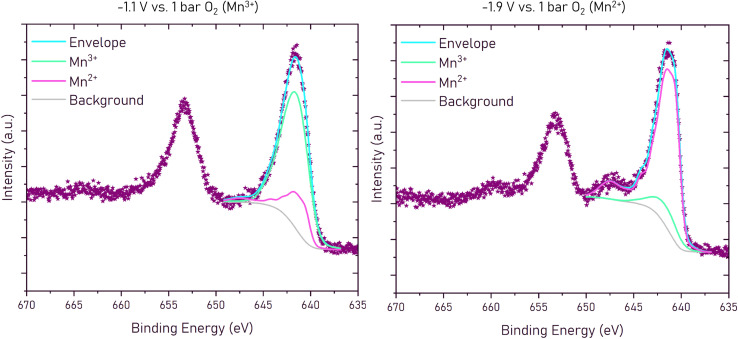
Mn 2p spectra of LSCrMn in UHV at 400 °C at −1.1 and −1.9 V against 1 bar O_2_. Two different components (Mn^3+^ and Mn^2+^) can be fitted to the spectra. At −1.1 V against 1 bar O_2_, Mn^3+^ is the main component of the spectra (green) and at −1.9 V against 1 bar O_2_, Mn^2+^ is the main component of the spectra (pink). The envelope (as the total fit of the spectra) can be seen in blue and the background in grey. Data points from the measurement are shown in purple.

To further validate the conclusions drawn on the redox chemistry, XPS measurements were performed on LSCrMn thin films at 400 °C in UHV. The oxygen chemical potential in the LSCrMn working electrode was controlled by applying a voltage *vs.* a counter electrode with a Fe/FeO buffer of a constant oxygen chemical potential. The Fe/FeO equilibrium has a Nernst potential of −1.141 V *vs.* 1 bar O_2_ at 400 °C,^35^ so we can use the relation U *vs.* O_2_ = U *vs*. CE −1.141 V. Thus, even without a gas phase in the XPS chamber, electrochemical oxygen activity control (EXACT = Electrochemical oXygen Activity ConTrol) of the LSCrMn film is possible. The sample is sketched in the experimental section and experimental details are given by Nenning *et al.*^36^ In accordance with our interpretation, only the Mn 2p XPS peaks showed clear changes with varying bias. Examplary Mn 2p XPS spectra and peak fitting results for −1.1 and −1.9 V *vs.* 1 bar O_2_ are shown in Fig. 7, with detailed fitting parameters and raw data provided as VAMAS files in the SI.

Peak fitting was performed using CasaXPS. The Mn 2p fitting was adapted from Biesinger *et al.*^37^ and Ilton *et al.*^38^ The peak model is a superposition of Mn^3+^ and Mn^2+^ fingerprint patterns, so that only the total position and the Mn^2+^/Mn^3+^ fraction remained as fit parameters. These constraints to the fit model avoid overparametrisation. In XPS measurements *ca.* 75% of the signal come from the topmost 2 nm, so we are actually looking at near-surface oxidation states which may be more reduced than the average bulk. However, recent studies on SrTi_0.6_Fe_0.4_O_3−*δ*_ (STF) suggest that at least for this perovskite, surface and bulk oxidation states do not differ drastically.^36^ It is therefore deemed plausible that the changes in surface oxidation states measured in this work are also representative of changes in bulk oxidation states.

**Fig. 8:**
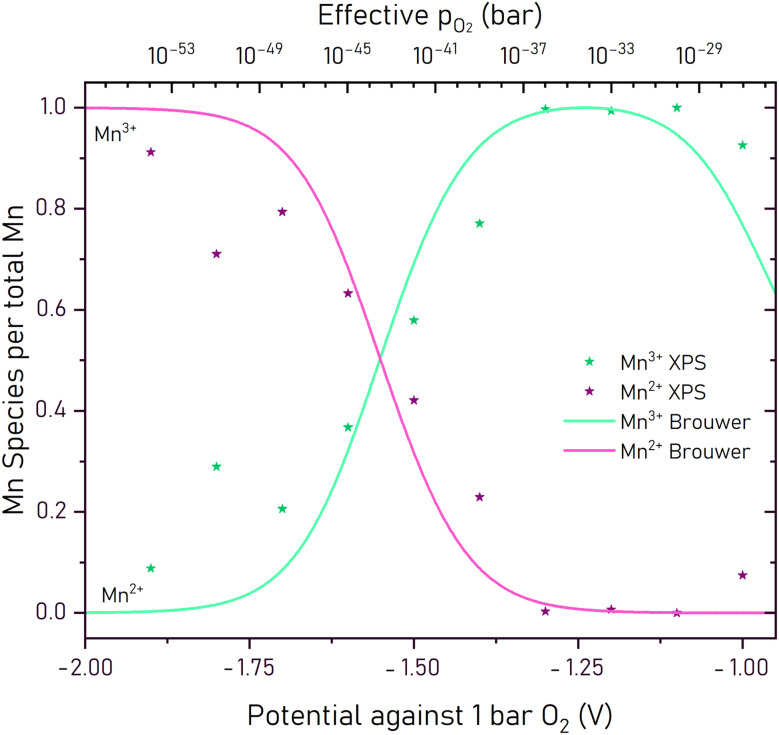
Mn^3+^ and Mn^2+^ concentrations obtained from EXACT-XPS measurements as a function of the potential *versus* the oxygen reference (symbols). Those are compared to calculated values from the Brouwer defect model in LSCrMn thin films (lines).

The Mn^3+^ and Mn^2+^ concentrations obtained from the EXACT-XPS measurements are shown in Fig. 8 in dependence of the voltages *vs.* 1 bar O_2_. In the range from −0.95 V to −1.95 V the Mn^3+^/Mn^2+^ ratio changes from virtually no Mn^2+^ to almost exclusively Mn^2+^. This change is accordingly accompanied by a strong decrease of the Mn^3+^ concentration. Mn^3+^/Mn^2+^ ratios close to unity are met around circa −1.53 V. In Fig. 8 these changes are compared with the predictions from the Brouwer diagram deduced below. The close match between the experimentally determined and modeled Mn valence states supports the reliability of the defect chemical model. In our case, we assume that the thin-film geometry, intermediate measurement temperatures, and the oxygen-conducting properties of LSCrMn enable a fast equilibrium between surface and bulk oxygen chemical potentials. This minimizes any differences between the two regions. Additionally, the very good agreement among the oxidation states obtained from XPS, electrochemical capacity measurements, and defect-chemical modeling suggests that the observed surface states also reflect the bulk conditions during steady-state operations. We therefore assume that for LSCrMn bulk and surface oxidation states are very similar. However, some minor thermodynamic shifts due to differences between surface and bulk may still be present.

Within the relevant potential range from −1.2 V to −1.8 V *vs.* 1 bar O_2_, no detectable change in the Cr oxidation state was observed. The Cr XPS spectra and interpretation of the data is shown in the SI 2.

## 3 Defect chemical model (Brouwer diagram)

The analysis of the experimental data showed that charge/discharge curves reflect the progressive reduction of Mn from Mn^4+^ to Mn^3+^ and subsequently to Mn^2+^. This sequential reduction causes significant charge storage as each Mn redox reduction step also implies an introduction of oxygen vacancies. To further understand the characteristics and behavior of the material, the experimental data is now used to develop a bulk defect model for LSCrMn. The main defect chemical charge carriers taken into account are oxygen vacancies 
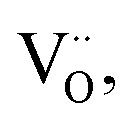
 electron holes h· which are synonymous for Mn^4+^ or 
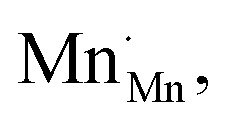
 electrons *e*^−^ (Mn^2+^, 
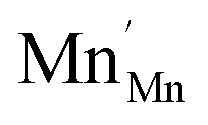
), 
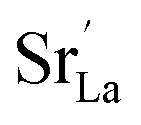
 acceptors and trap states. Cr ions are assumed to be stable (3+). The exact nature of the redox states or trap states, being relevant at very negative potentials below −1.8 V, is not yet fully clear. They could correspond either to singly charged oxygen vacancies 
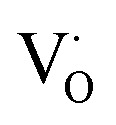
 or Cr^2+^
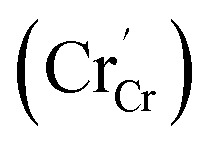
 appearing at very low *p*_O_2__ regions. In order to be specific we assume oxygen vacancies as electron traps, *i.e.* existence of 
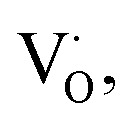
 but all main considerations remain valid also for 
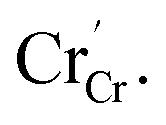
 The relations between the three Mn redox states can be expressed in Kröger–Vink notation as a disproportionation reaction8
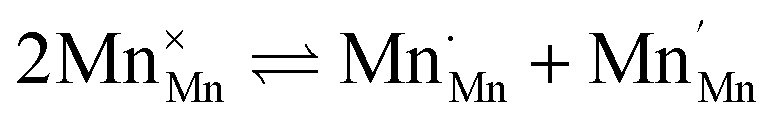
The oxygen exchange reaction (incorporation) in the reducing regime reads:9
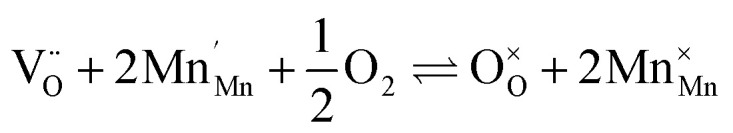
and the trap reaction is given by:10
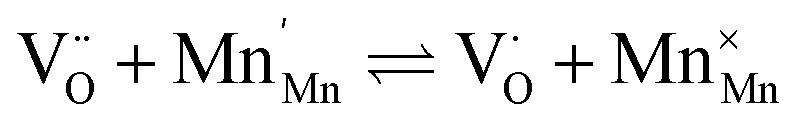
Thus, six defect species are taken into account, and six equations with six unknown variables have to be defined. These equations reflect charge balance, site constraints, and defect equilibria relevant to oxygen vacancies and the redox behavior of manganese. The charge neutrality in the system is given by11
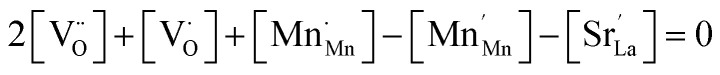
with [] indicating the concentration normalized to a unit cell. In any case 
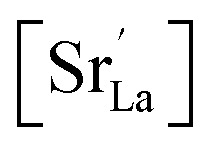
 is fixed to 0.5 in our study. The total positive charge from doubly or singly charged oxygen vacancies 
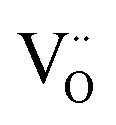
 and Mn^4+^ species 
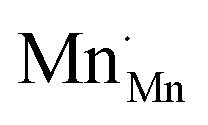
 must be balanced by the negative charge from Mn^2+^ ions 
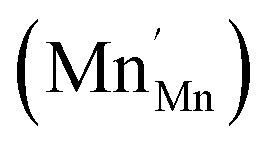
 and the Sr dopants. Mn disproportionation is represented by12
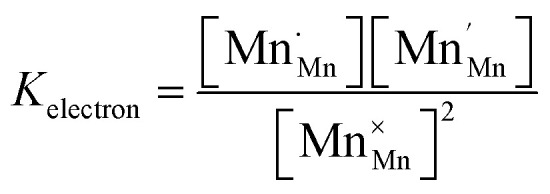
and the equilibrium of oxygen exchange leads to13
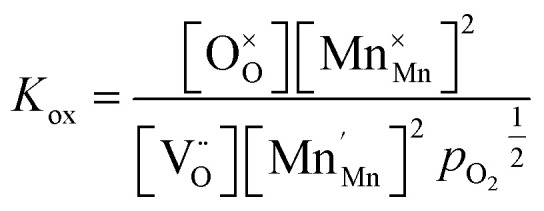
The effective oxygen partial pressure *p*_O_2__ is normalized to 1 bar O_2_. The equilibrium constant *K*_trap_ of the trapping of charge carriers at oxygen vacancies, forming singly charged vacancies, is given by:14
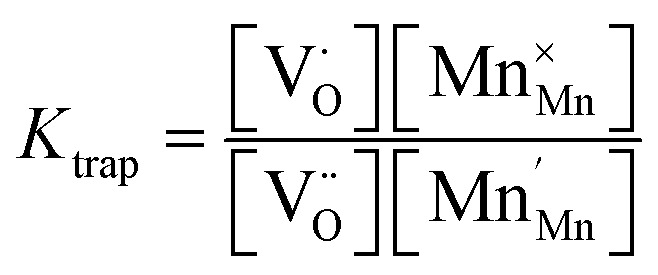


From the chemical formula of LSCrMn (La_0.5_Sr_0.5_Cr_0.2_Mn_0.8_O_3−*δ*_), the total manganese concentration of any oxidation state is fixed at 0.8 per formula unit. This site constraint for manganese leads to15
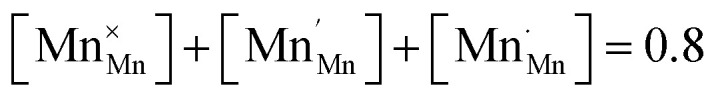


In the ideal perovskite structure, there are three oxygen sites per formula unit. The oxygen lattice occupancy is thus quantified by16
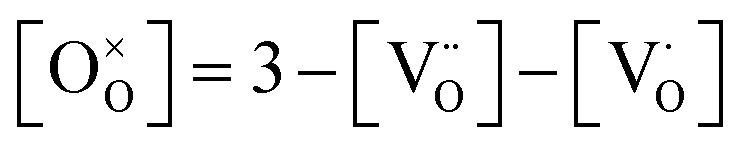


From these six equations, the defect concentrations can be calculated as a function of the oxygen partial pressure, which can further be assigned to a certain overpotential against 1 bar O_2_.

The defect model possesses three degrees of freedom or fit parameters: the equilibrium constants *K*_ox_, *K*_electron_, and *K*_trap_, since the composition is fixed to a Sr doping concentration of 0.5 and a Mn concentration of 0.8. These parameters control the chemical potentials or equivalently the oxygen partial pressure where redox processes take primarily place, *i.e.* where the majority defect transitions occur.

In the first step, the system of equations was solved numerically, to obtain an expression for the concentration of lattice oxygen 
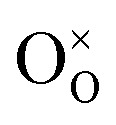
 as a function of *p*_O_2__ or oxygen chemical potential µ_O_. This concentration 
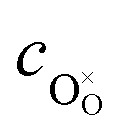
 is particularly important, as it is directly related to the oxygen non-stoichiometry in the material and allows the calculation of the chemical capacitance *C*_chem_*via*:17
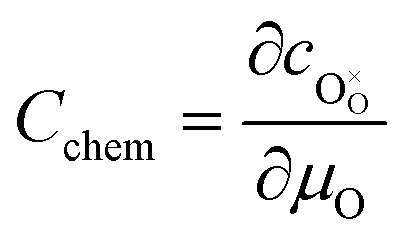


This numerically calculated *C*_chem_ was then quantitatively compared to the experimentally measured capacitance data (presented above). The three constants *K*_ox_, *K*_electron_, and *K*_trap_ were adapted to reproduce the experimental curve of *C*_chem_ from EIS, enabling the model to match the observed defect transitions. In Table 4 the values of the three fit parameters are summarized and compared to literature values of La_0.8_Sr_0.2_MnO_3_ (LSM) and La_0.9_Sr_0.1_CrO_3_ (LSCr). To be able to compare these values a few things have to be considered. For this paper, we write the oxygen exchange expressed with electrons, and the hole/electron formation as two separate equation, as seen in eqn (8) and (9). The reference sources calculated enthalpy and entropy values by considering the oxygen exchange with regard to the electron holes (see eqn (18)), which is a combination of the above two. So to compare the acquired data with literature values, the *K*_ox_ values were adequately converted.18
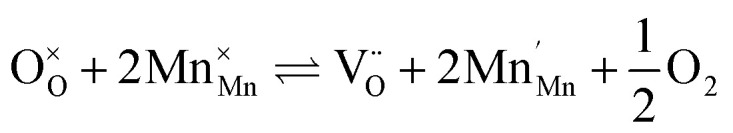


**Table 4:** Values for the equilibrium constants *K*_ox_, *K*_electron_ and *K*_trap_ and reaction enthalpies (Δ*H*^0^_x_) and entropies (Δ*S*^0^_x_) (oxygen exchange (x = ox), electron/hole formation (x = electron) and trap formation (x = trap)) for La_0.5_Sr_0.5_Mn_0.8_Cr_0.2_O_3_ (LSCrMn), La_0.8_Sr_0.2_MnO_3_ (LSM) and La_0.9_Sr_0.1_CrO_3_ (LSCr) at 400 °C. The values for LSM and LSCr were calculated from literature data and for experimentally obtained for LSCrMn

	LSCrMn	LSM	LSCr
Δ*H*^0^_ox_ (kJ mol^−1^)	—	−302.5 ± 15.3	−303.1
Δ*S*^0^_ox_ (J mol^−1^ K^−1^)	—	−114.5 ± 11.7	−100.5
Reference	This study	Nowotny^39^	Mizusaki^40^
*K*_ox_	2.07 × 10^−23^	3.21 × 10^−18^	5.36 × 10^−19^
Δ*H*^0^_electron_ (kJ mol^−1^)	—	0 ± 0	—
Δ*S*^0^_electron_ (J mol^−1^ K^−1^)	—	−25.1 ± 6.6	—
Reference	This study	Nowotny^39^	—
*K*_electron_	4.56 × 10^−6^	4.89 × 10^−2^	—
Δ*H*^0^_ox_ (kJ mol^−1^)	—	—	—
Δ*S*^0^_ox_ (J mol^−1^ K^−1^)	—	—	—
Reference	This study	—	—
*K*_trap_	2.54 × 10^−5^	—	—

**Fig. 9:**
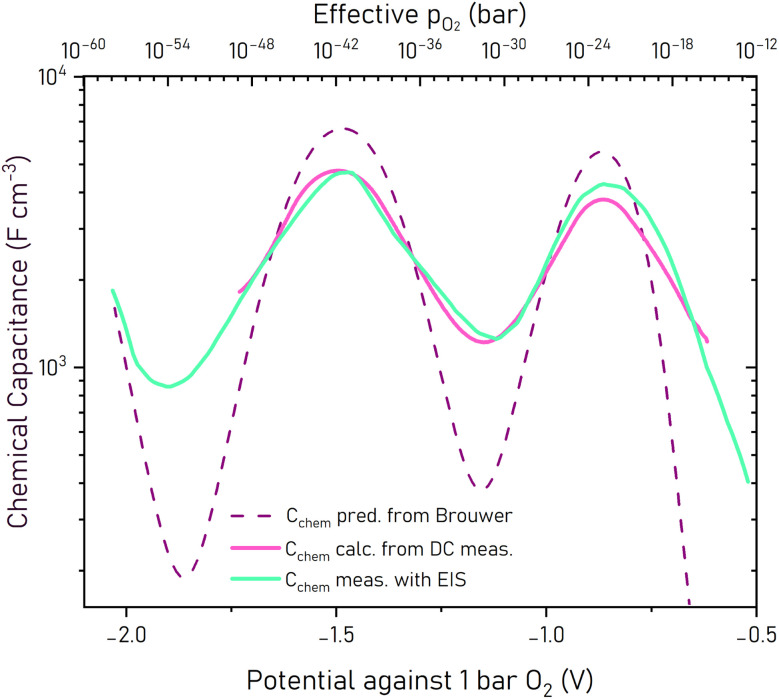
Comparison of the experimentally (EIS) measured chemical capacitance *C*_chem_ and values predicted from the defect model (Brouwer) *via* the derivative of lattice oxygen concentration with respect to the potential. The model reproduces the peak positions well, indicating that the defect chemical equilibria are accurately captured. Also *C*_chem_ calculated from the charge curves is given.

Deviations to literature data of *K*_ox_ and *K*_electron_ values at 400 °C are not surprising owing to the different composition and also due to the given literature values of Δ*H*^0^ and Δ*S*^0^ being determined at temperatures in the range of 1000 °C. Moreover, defect thermodynamic data of thin films may differ from those of bulk materials.^16^ Taking this into account, our values are in very reasonable ranges. The corresponding *C*_chem_*vs. p*_O_2__ curve is shown in Fig. 9 (“*C*_chem_ pred. from Brouwer”). Alternatively, one may also directly fit the oxygen concentration (scaled by appropriate prefactors) to the charge *vs.* voltage curve obtained from the DC measurement; similar values result. However, the impact of the three free parameters of our model, namely the three equilibrium constants, is much better visible in the differential (*i.e. C*_chem_) representation, than in the integral one (charge/discharge curve). Specifically, in the *C*_chem_ curve these constants directly determine the position of capacitance peaks, whereas in the DC curve, they show up only as inflection points.

As shown in the comparison of *C*_chem_ values predicted from the Brouwer diagram and measured by EIS (Fig. 9), our defect model can qualitatively reproduce the *C*_chem_*vs. p*_O_2__ curve shape. In particular the positions of minima and maxima align well. While the peaks are a bit slimmer and the slopes steeper in the “predicted” curve, the peak area fits well. This confirms that the defect model, with its three fit parameters *K*_ox_, *K*_electron_, and *K*_trap_, successfully captures the redox transitions and oxygen vacancy equilibria of the LSCrMn system. These constants primarily influence the µ_O_-position of the peaks—i.e., the potential or effective oxygen partial pressure—without significantly altering the peak shapes. We may also compare the chemical capacitance obtained from *C*_chem_ with that obtained from the DC curve by derivation (*i.e.* dQ/dU). This is shown in Fig. 9 as well (“calc. from DC meas.“). The locations of the minima and maxima across the *p*_O_2__ range correspond very well to the predictions from both Brouwer and EIS measurements. And since the fit parameters of our model primarily govern the position of the *C*_chem_ maxima, which agree in both experiments, we are confident in the equilibrium constants obtained from both fits.

**Fig. 10:**
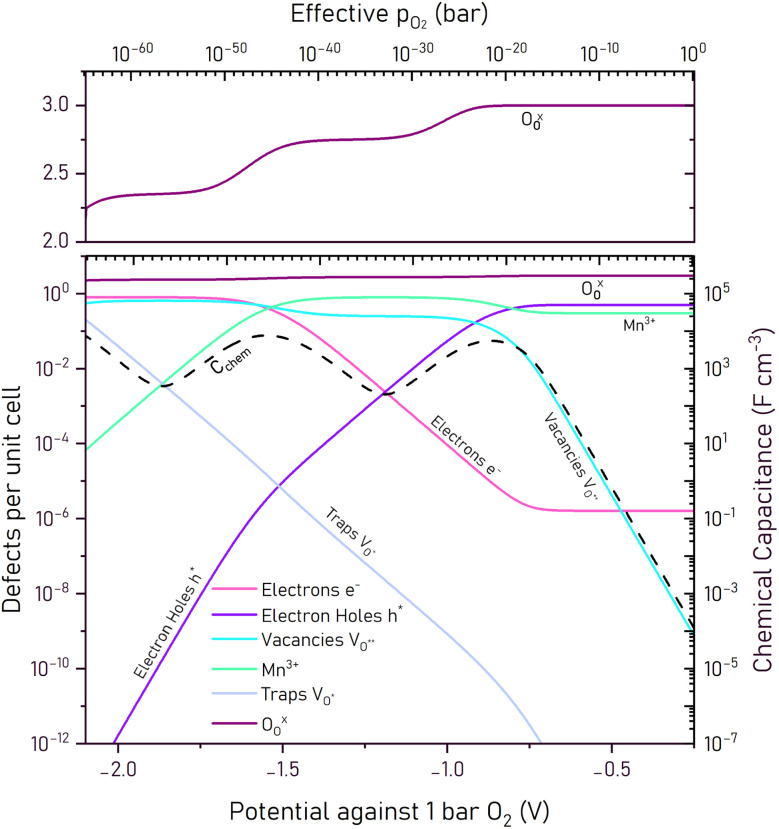
Brouwer diagram showing calculated defect concentrations in bulk La_0.5_Sr_0.5_Cr_0.2_Mn_0.8_O_3−*δ*_ (LSCrMn) at 400 °C as a function of oxygen partial pressure. The diagram includes electron holes 
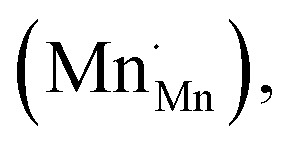
 electrons 
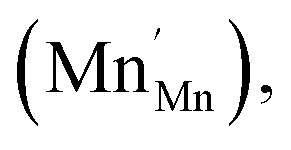
 Mn^3+^
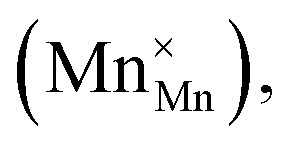
 doubly and singly charged oxygen vacancies 
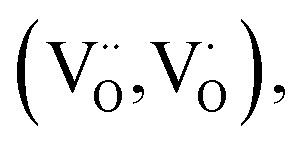
 and lattice oxygen 
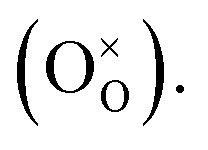
 Defect concentrations are based on thermodynamic modeling and fitted EIS measurements. The chemical capacitance curve, previously shown as “*C*_chem_ pred. from Brouwer” in Fig. 9 is shown as the dotted black line in the diagram.

Based on the fitted parameters *K*_ox_, *K*_electron_, and *K*_trap_, the complete Brouwer diagram was calculated, showing the concentration of all relevant defect species as a function of potential and oxygen partial pressure at 400 °C (Fig. 10).

This Brouwer diagram illustrates the equilibrium concentrations of point defects as a function of the effective oxygen partial pressure *p*_O_2__ (upper *x*-axis, logarithmic scale) and the corresponding potential against 1 bar O_2_ (lower *x*-axis, linear scale). The *y*-axis shows the defect concentrations on a logarithmic scale as defects per unit cell. At high potentials, above the Mn^4+^/Mn^3+^ redox potential, electron holes are the dominant charge carriers. In this regime, charge compensation for the substitution of La^3+^ with Sr^2+^ on the A-site is primarily achieved by oxidation of the manganese into the +IV oxidation state, while the rest exists as Mn^3+^. As a result, the Mn^3+^ concentration remains relatively constant, while the hole concentration is high.

With a decrease in oxygen partial pressure or potential, the concentration of oxygen vacancies increases in this region, reflecting a gradual oxygen release from the perovskite lattice. As the potential further decreases and approaches the Mn^4+^/Mn^3+^ redox potential, Mn^4+^ is progressively reduced to Mn^3+^ and the hole concentration no longer compensates most of the acceptor doping. The oxygen vacancy concentration rises. This transition from hole to vacancy compensation causes the first peak in the chemical capacitance and equivalently the first plateau in the charge/discharge curve. At −0.91 V against 1 bar O_2_, corresponding to an effective oxygen partial pressure of approximately 5 × 10^−25^ bar, oxygen vacancies become the dominant defect species.

With further decrease in potential, Mn^3+^ begins to reduce to Mn^2+^ and thus increases the electron concentration. This transition from Mn^3+^ to Mn^2+^ is responsible for the second peak in the chemical capacitance and the second charging plateau. It is also observed in the XPS experiments and the transition potential suggested by the Brouwer diagram is in excellent agreement with the XPS data (see Fig. 8). At even more reducing potentials, trap states begin to become relevant. These may correspond to partially ionized oxygen vacancies or the onset of a reduction of the chromium from Cr^3+^ → Cr^2+^. Their contribution, however, only becomes significant below −2.07 V and this process is visible only as the onset of a third peak in the *C*_chem_ curve, or equivalently the onset of a sharp decrease in the charge/discharge curve.

## 4 Experimental

### 4.1 Sample Preparation and Analysis

Yttria stabilized zirconia (YSZ) single crystals (MaTeck GmbH, Germany, and Crystec, Germany) were used as the substrate and electrolyte of the samples. The single crystals were polished on one side and had a lattice orientation of (100). The dimensions were 10 mm × 10 mm × 0.5 mm. Each substrate was cleaned in a three-step process with Extran (Merck, Germany), ethanol, and de-ionized water. For every cleaning step, the samples were placed in a 50 °C ultrasonic bath for 30 minutes. Afterwards, they were annealed at 1300–1350 °C in air for 6 h.

**Fig. 11:**
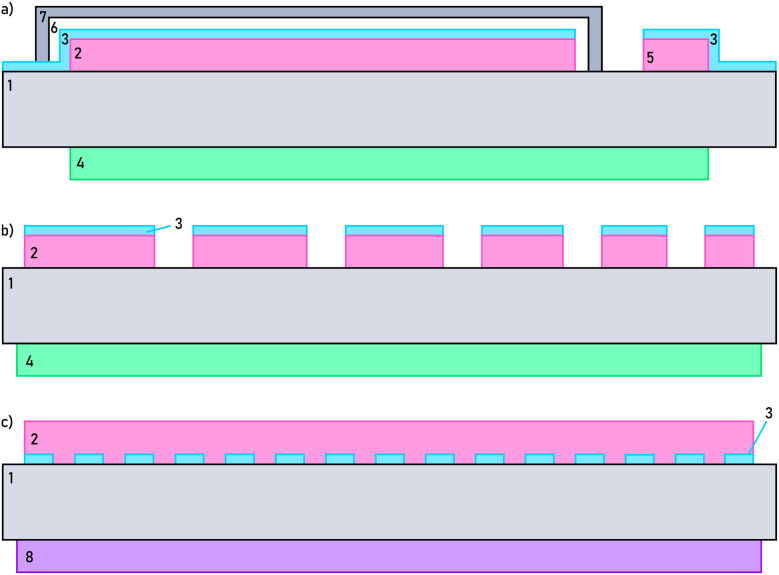
Schematic sketch of the cross-section of the (a) half-cell sample for charge/discharge curves, (b) microelectrode sample for impedance measurements and (c) EXACT-XPS sample. (1) YSZ single crystal substrate/electrolyte, (2) LSCrMn WE, (3) Pt/Ti current collector, (4) LSC CE, (5) LSCrMn RE, (6) ZrO_2_ sealing layer, (7) Glass sealing layer, (8) Pt/GDC10/Fe_2_O_3_ CE. A photograph of the half cell and the microelectrode samples, for a better, real-life understanding of the architectures, can be seen in the SI 3.

For the half-cell samples used in charge/discharge experiments, porous LSC was used as the counter electrode (CE). The corresponding 300 nm LSC layer was deposited on the non-polished side of the single crystal *via* pulsed laser deposition. A 300 nm thick layer of LSCrMn was then deposited on the polished side of the YSZ single crystal and a deposition mask was used to create two separate electrode areas. All thin film depositions were done using a KrF excimer laser (Complex Pro 201F, 248 nm) with parameters given in Table 5. The larger part (0.27 cm^2^) was used as the working electrode (WE) and the smaller part (0.04 cm^2^) as the reference electrode. After deposition, a planar current collector was placed on top, consisting of a sputtered 10 nm titanium layer and a 100 nm platinum layer. The WE was then sealed with a PLD-deposited 500 nm thick ZrO_2_ layer and an additional glass seal. The glass seal was prepared from a glass paste (Schott AG, Germany) and first melted at 700 °C in an oven with slow heating and cooling rates (1 °C min^−1^), then re-melted in the measurement set-up at 700 °C for 15 minutes, before being cooled to the desired temperature. Fig. 11a shows a sketch of the sample architecture with all components.

**Table 5:** PLD Parameters used for depositing the LSCrMn and LSC thin films. For all depositions, the target–substrate distance was set to 6 cm, the pulse frequency to 10 Hz. The deposition rate was determined accordingly with a Qartz Micro Balance (QMB) beforehand

Material	*p*(O_2_) (mbar)	Temperature (°C)	Fluence (J cm^−2^)
LSCrMn	4 × 10^−2^	700	1.0
LSC (porous)	4 × 10^−1^	450	1.0

For the microelectrode samples used in electrochemical impedance spectroscopy measurements, porous LSC (La_0.6_Sr_0.4_CoO_3−*δ*_) was used as the counter electrode (CE) as well. For the working electrode (WE), a 300 nm thick layer of LSCrMn (La_0.5_Sr_0.5_Cr_0.2_Mn_0.8_O_3−*δ*_) was deposited on the polished side of the YSZ single crystal. Deposition parameters are listed in Table 5. After deposition, a current collector was placed on top of the MIEC working electrode. It again consists of a 10 nm titanium layer and a 100 nm platinum layer, grown by DC magnetron sputtering. Microelectrodes were fabricated *via* photolithography and ion beam etching and for the electrochemical impedance spectroscopy measurements microelectrodes with a diameter of 300 µm were used. Fig. 11b shows a sketch of the sample architecture with all components. The thin-film electrode geometry was intentionally chosen to enable well-defined electrochemical boundary conditions, minimize transport limitations, and allow quantitative extraction of defect-chemical parameters. While thicker electrodes may experience additional kinetic constraints (*e.g.*, oxygen chemical diffusion or grain boundary effects), the fundamental redox mechanism, namely reversible oxygen vacancy formation coupled to Mn reduction, is therefore expected to remain valid. However, different thermodynamic data of the defect chemical reaction may induce some potential shifts and further experimentation is required to see how the electrode behaves in terms of capacity, mechanical stability, and utilization in thicker electrodes.

For the samples used in X-ray photoelectron spectroscopy (XPS) measurements, a porous Pt/GDC10/Fe_2_O_3_ electrode (GDC10 = Ce_0.9_Gd_0.1_O_2−*δ*_) was used as the CE. A paste consisting of 80 wt% GDC10 and 20 wt% Fe_2_O_3_ was spin-coated on the unpolished side of the YSZ single crystal, and then dried at 120 °C. Afterwards, a Pt paste was brushed on top of the dried GDC/Fe_2_O_3_ layer, as a current collector. As a last step, the CE was sintered at 1050 °C in air for 3 h. Under XPS measurement conditions a Fe/FeO mixture is formed in the counter electrode, see below, and thus has a fixed oxygen chemical potential corresponding to 6.78 × 10^−35^ bar O_2_ at 400 °C. Hence, it allows well-defined experiments also in the ultra high vacuum (UHV) of XPS instruments. On the polished side, a 10/100 nm Ti/Pt layer was sputtered and lithographically patterned into a 15/35 µm grid. For the WE, a 300 nm thick layer of LSCrMn was deposited on top of this grid. Fig. 11c shows a sketch of the sample architecture with all components.

The deposited LSCrMn thin films were characterized using X-ray diffraction (XRD), atomic force microscopy (AFM), and scanning electron microscopy (SEM) measurements. XRD measurements were conducted in the range of 5°–90° using an Empyrean X-ray diffractometer (Malvern Panalytical, U.K.) in a Bragg–Brentano geometry. AFM images of the LSCrMn sample surface were taken with a Nanoscope V multimode setup (Bruker, USA). SEM images were aquiered on a Quanta 250 FEG (FEI, USA). Although all samples have some electric conductivity, a thin layer of gold was sputtered to achieve an even conductivity and ensure good SEM image results.

### 4.2 Electrochemical impedance spectroscopy and DC measurements

For the charge/discharge cycles, a measurement set-up with a Keithley 2000 Digital Multimeter and a Keithley 2600 Source Meter Unit (Tectronix, Inc., USA) was used. The sample was placed on a Pt sheet inside the furnace, and the WE and RE were connected with a Pt–Ir needle each, while the CE was connected *via* a platinum sheet. The RE is used to reduce the contribution of the overall overpotential to the relevant voltage. The temperature inside the furnace was controlled by a Eurotherm 3216 (Eurotherm, Germany) temperature control system. The measurements were conducted in a 1% oxygen in *N*_2_ atmosphere, see above, and voltages given in the paper are values recalculated to 1 bar O_2_ by Nernst's equation. A constant current of 10 µA (*i.e.* 37 µA cm^−2^) was applied, the electrode was therefore charged, and the voltage was measured between counter and working electrode as well as between reference and working electrode. However, it turned out that the difference between the voltages *vs.* the CE and the RE was mostly very small (generally < 30 mV). Once a certain cutoff voltage was reached between working and counter electrode, the current was inverted and the electrode was discharged.

For the electrochemical impedance measurements, an Alpha-A High Performance Frequency Analyzer with an Electrical Test Station POT/GAL 30V/2A (Novocontrol Technologies GmbH & Co. KG, Germany) was used. The sample was placed with the CE on a Pt sheet inside the furnace, and a 300 µm microelectrode (WE) was connected by a Pt–Ir needle. The temperature inside the furnace was controlled by a Eurotherm 3216 (Eurotherm, Germany) temperature control system. The measurements were conducted in a 1% oxygen in *N*_2_ atmosphere at 400 °C. The impedance was measured in the frequency range of 1 MHz to 4 mHz with 10 data points per decade. For all measurements, a voltage from 0 to up to −2000 mV was applied, with bias steps of either 50 or 100 mV. Voltages given in the paper are recalculated to 1 bar O_2_*via* Nernst's equation (see eqn (1)), *i.e.* are from −0.07 to −2.07 V. Six charge/discharge cycles were measured at each temperature, with the cycle starting at −0.07 V and decreasing to −2.07 V and then increasing to −0.07 V again.

### 4.3 XPS measurements

To study the effect of charging/discharging on the Mn oxidation state, XPS measurements using the EXACT (EXACT = Electrochemical oXygen Activity ConTrol) approach were performed, as described in detail by Nenning *et al.*^36^ The following is a short explanation of the EXACT-XPS method, for more details the reference should be considered. The EXACT method allows to control and modulate the equivalent *p*_O_2__ that defines µ_O_ in the WE while simultaneously keeping the sample at UHV conditions to enable XPS measurements. It is based on an oxygen ion buffering CE that is pinned at a defined oxygen activity *via* the Fe/FeO phase equilibrium. Under the UHV conditions present inside the XPS chamber, no net reaction of the electrodes with the surrounding gas atmosphere occurs, as long as the oxygen activity of the sample is kept sufficiently low. The sample then practically behaves like an oxygen ion battery, and oxygen ions can be reversibly transported from one electrode to the other. As the oxygen activity of the CE is well defined by the phase equilibrium, the oxygen activity of the WE can be controlled by a voltage U with respect to the CE. Please note that without DC current overpotentials are absent and the applied voltage is precisely proportional to the µ_O_ difference between WE and CE. Following Nernst's equation, the effective oxygen partial pressure 
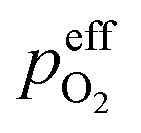
 (*i.e.* the oxygen activity) in the WE is then given by:19
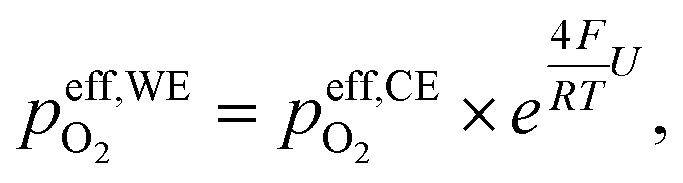
where20
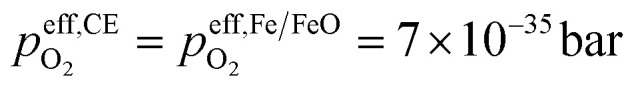
XPS measurements were carried out in a PHI Versaprobe 3 XPS spectrometer. The samples were mounted on a customized sample holder which allowed for electrical contacting of the sample by Pt–Ir needles and heating with a resistive heater. Electrochemical measurements were performed with a Bio-Logic SP-200 electrochemical test station at 400 °C. The temperature was controlled using a pyrometer, which was calibrated at the beginning of the measurement using the known temperature dependency of the resistivity of YSZ.

To establish the necessary Fe/FeO equilibrium in the CE, a conditioning step (0.24 mA, 12 min) was carried out prior to the measurements. During this, a controlled amount of oxygen was removed from the CE, partially reducing the present iron oxide to metallic iron. The measurement then consisted of first a stepwise decrease of the applied bias from 500 to −800 mV (*vs.* Fe/FeO) with a step size of 100 mV, followed by a stepwise increase back to 500 mV. XPS spectra of O 1s, Mn 2p, Cr 2p, La 3d, and Sr 3d were recorded at each potential step using a 50 W monochromated Al K*α* source with a 200 µm spot size. Survey scans were recorded with a pass energy of 140 eV, detailed element spectra with a pass energy of 27 eV, with the analyzer tilted 45° relative to the surface normal.

## 5 Conclusions

La_0.5_Sr_0.5_Cr_0.2_Mn_0.8_O_3−*δ*_ was investigated as potential anode materials for application in oxygen ion batteries. The aim was to assess its electrochemical performance, redox stability, and defect chemistry across a wide range of oxygen chemical potentials/voltages *vs.* 1 bar O_2_. Overall, the results show that LSCrMn can store very substantial amounts of charge *via* the Mn B-site cation and its reversible redox mechanisms from Mn^4+^ to Mn^3+^ and subsequently to Mn^2+^, while maintaining structural integrity down to potentials of about −2 V *vs.* O_2_, where pure LSM perovskites would already decompose.

DC cycling measurements on thin film electrodes revealed two distinct charge/discharge plateaus, corresponding to the stepwise reduction of Mn^4+^ to Mn^3+^ (−0.8 V plateau) and subsequently to Mn^2+^ (−1.4 V plateau). The course of the DC curve remained stable across more than 70 cycles, with an average total discharge capacity of 938 mA h cm^−3^. The observed coulomb efficiency of 92% suggests some irreversible losses, possibly due to a leaky glass seal layer. Voltage dependent electrochemical impedance spectroscopy further confirmed these redox steps. The spectra were quantitatively fitted by using a modified Randles' circuit, and the measured chemical capacitances showed maxima and minima at the potentials expected from the charge/discharge curves. Also ionic resistivities, in the range of 4 × 10^−5^ Ω cm at 400 °C could be estimated in the relevant voltage range of high oxygen non-stoichiometry.

To understand the underlying defect chemistry, a bulk defect model was established, based on the involved charge carriers and defect equilibria. After fitting three key constants (*K*_ox_, *K*_electron_, and *K*_trap_) to the experimentally obtained data, the model was able to reproduce the measured chemical capacitance *vs.* potential with good agreement. The calculated Brouwer diagram reflects the expected defect changes with decreasing *p*_O_2__: starting hole-dominated at high potentials, we get oxygen vacancy formation and Mn^4+^ → Mn^3+^ reduction towards lower potentials, and eventually Mn^2+^ and electron-dominated conduction at very low oxygen chemical potentials. The defect transitions captured by the model were further validated by EXACT-XPS measurements. While XPS is surface-sensitive, the good agreement with modeled Mn^3+^/Mn^2+^ concentrations across the relevant potential range indicate that surface oxidation states reflect bulk behavior in LSCrMn to a very reasonable degree. This supports the assumption, that only the manganese on the B-site of the perovskite undergoes redox transitions.

Thus, our study reveals that La_0.5_Sr_0.5_Cr_0.2_Mn_0.8_O_3−*δ*_ is a highly stable MIEC perovskite, capable of being used as an anode material in the low-*p*_O_2__ regime needed for oxygen ion batteries. The reversible Mn redox activity, combined with structural stabilization by Cr, enables both high capacity and a large operation voltage down to almost −2 V. The work also exemplifies how specific perovskite B-site doping strategies can extend the usable redox window of perovskite oxides into new application fields.

## Author contributions

Barbara Wagner: conceptualization, validation, methodology, formal analysis, investigation, data curation, writing – original draft, writing – review & editing, visualization, project administration. Alexander Schmid: conceptualization, methodology, validation, resources, writing – review & editing, supervision. Stanislaus Breitwieser: EXACT-XPS: validation, methodology, formal analysis, investigation, writing – review & editing. Andreas Nenning: EXACT-XPS: methodology, writing – review & editing. Jürgen Fleig: resources, writing – review & editing, supervision, funding acquisition.

## Conflicts of interest

There are no conflicts of interest to declare.

## Note added after first publication

This article replaces the version published on 20th May 2026. Eqn 10 has been corrected.

## Supplementary Material

TA-014-D6TA00585C-s001

## Data Availability

Supplementary information (SI): the structure of the supporting XPS data sets is explained in the supporting pdf file. The shown XPS data collected and presented for this study are available in Vamas format (readable *e.g.* by CasaXPS) in the SI. See DOI: https://doi.org/10.1039/d6ta00585c.
