# Supplementary material for: The redox chemistry of La0.5Sr0.5Cr0.2Mn0.8O3−δ and its application in high capacity anodes of oxygen ion batteries
Source: J Mater Chem A Mater. 2026 May 20;14(38):25240–54. doi: 10.1039/d6ta00585c (PMC13187869; doi:10.1039/d6ta00585c)
Supplement: TA-014-D6TA00585C-s001 [file TA-014-D6TA00585C-s001.zip › supporting information.pdf]

Supporting information for

The redox chemistry of $La_{0.5}Sr_{0.5}Cr_{0.2}Mn_{0.8}O_{3-\delta}$ and its application in high capacity anodes of oxygen ion batteries

Barbara Wagner, Alexander Schmid, Stanislaus Breitwieser, Andreas Nenning, Jürgen Fleig

Institute of Chemical Technologies and Analytics, Research Group for Electrochemical Energy Conversion, TU Wien, Getreidemarkt 9, 1060 Wien, Austria

Correspondence: barbara.wagner@tuwien.ac.at

1. Charge Transfer Resistance (R_{ct}) and Interfacial Capacitance (C_{int})

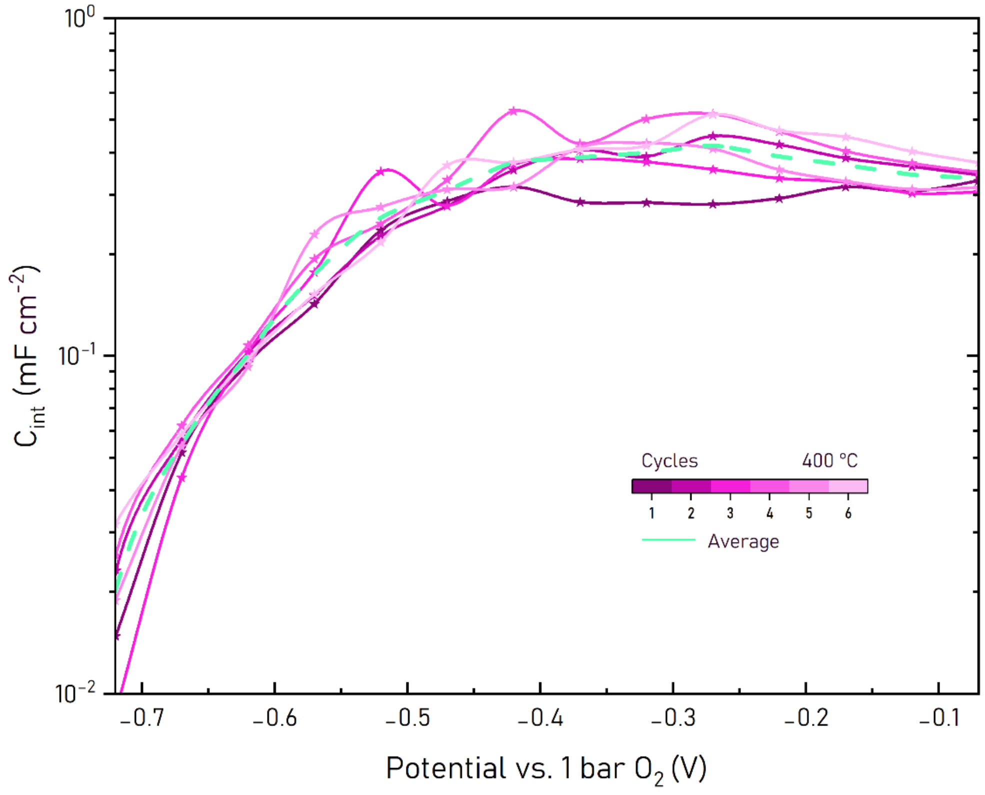

Supporting Figure 1: Interfacial capacitance of the microelectrode sample at 400 °C, calculated from fitted impedance spectra, shown as values across six cycles and the average across all six cycles.

The charge transfer resistance (R_{ct}) and interfacial capacitance (C_{int}) of the LSCrMn microelectrodes were obtained from impedance spectra measured at 400 °C. As mentioned in the main text, the $C_{dl}||R_{ct}$ feature is a medium frequency feature that dominates the spectra at low applied biases and shrinks drastically with increasing negative bias, before visually disappearing at an applied bias below - 0.72 V vs. 1 bar O_2. Applied circuit models are shown

in the main text in Figure 4c) (– 0.07 V to – 0.47 V) and Figure 4e) (– 0.52 V to – 0.72 V). All fitted values were normalized to the geometric electrode area of the microelectrode (7.1 × 10^{-4} cm^2).

Supporting Figure 1 shows C_{int} as a function of potential across six measurement cycles. C_{int} remains almost constant at around 0.4 mF cm^{-2} between – 0.07 V and – 0.47 V, followed by a sharp decrease to circa 0.02 mF cm^{-2} at – 0.72 V, indicating a potential-dependent change in interfacial capacitance under increasingly reducing conditions. The large value of 400 µF/cm2 in the low voltage range is far beyond of what one might expect from an electrochemical double layer, i.e. an electrostatic capacitor. Most probably some chemical charge storage at the interface is involved which seems to decrease strongly upon reducing conditions. Specific mechanisms causing this capacitance are unknown yet. However, large interfacial capacitances are also reported in literature for $La_{0.6}Sr_{0.4}CoO_{3-\delta}$ and $La_{0.6}Ba_{0.4}CoO_{3-\delta}$ [1].

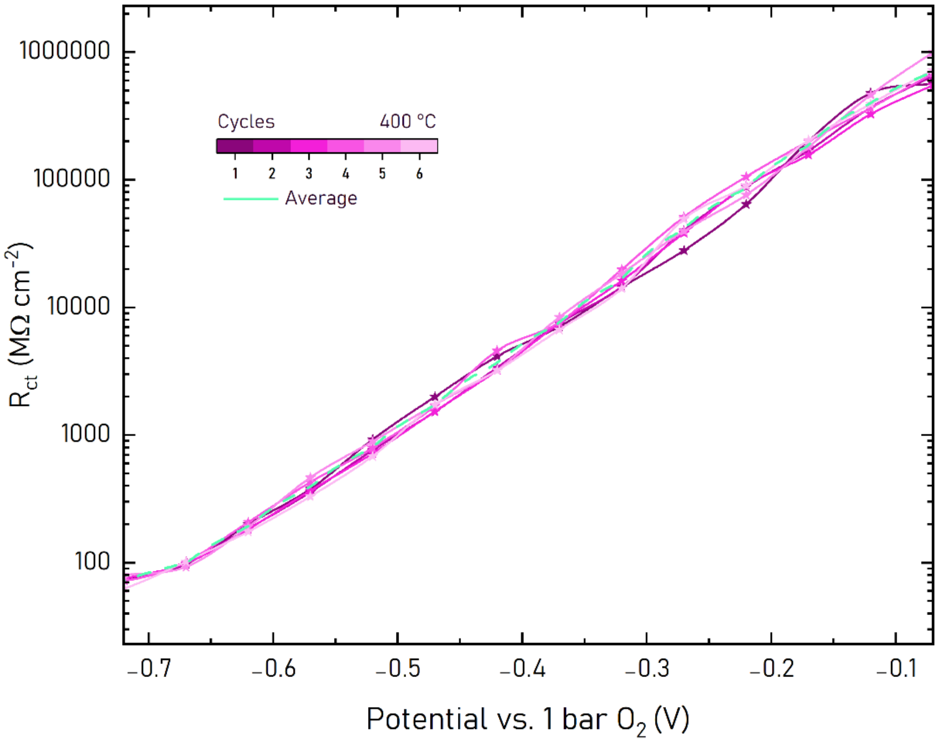

Supporting Figure 2: Charge transfer resistance of the microelectrode sample at 400 °C, calculated from fitted impedance spectra, shown as values across six cycles and the average across all six cycles.

Supporting Figure 2 shows the corresponding Rct values, which decrease from 7.2 × 10^{11} Ω cm^2 near open-circuit potentials to 1.3 × 10^{10} Ω cm^2 at – 0.35 V and 7.85 × 10^7 Ω cm^2 at – 0.72 V.

In the calculated Brouwer diagram (see main text Figure 10), the oxygen vacancy concentration drastically increases in the given voltage range and thus a substantial decrease of R_{ct} with more negative potentials is in accordance with the interpretation of R_{ct} as the charge transfer at the electrode/electrolyte interface.

2. XPS:

As written in the supporting information notice in the main text, the XPS raw data and the Mn 2p peak fit are provided as VAMAS files (readable i.e. with CASAXPS).

The measurements consisted of a step-wise decrease in voltage from -0.6 V down to -1.9 vs. 1 bar O_2 in 100 mV steps. This was followed by a step-wise re-oxidation of the sample back to -0.6 V vs 1 bar p_{O2}. XPS spectra of Cr 2p, O 1s, Mn 2p, Sr 3d and La 3d were recorded at each potential step. (For practical reasons, the voltage was switched during the survey measurement.)
The voltage of each step is given in the "Sample Identifier" row ("counter electrode conditioning" referees to the conditioning steps described in "UHV-based analytics with electrochemical oxygen activity control" [2] and corresponds to the most oxidizing conditions measured). Mn 2p was fitted using one fingerprint shape each for the 3+ and 2+ oxidation state, derived from [3][4]. Components and restrictions can be examined in the "Quantification Parameters", where the components "Mn 2p 3+ A-E" together create the Mn 3+ peak shape, while "Mn 2p 2+ A-H" create the Mn 2+ peak shape. (To display the peak shapes as shown in the manuscript got to "Options" -> "Tile Display" -> "Colours" and select "Combine Copm Index".)

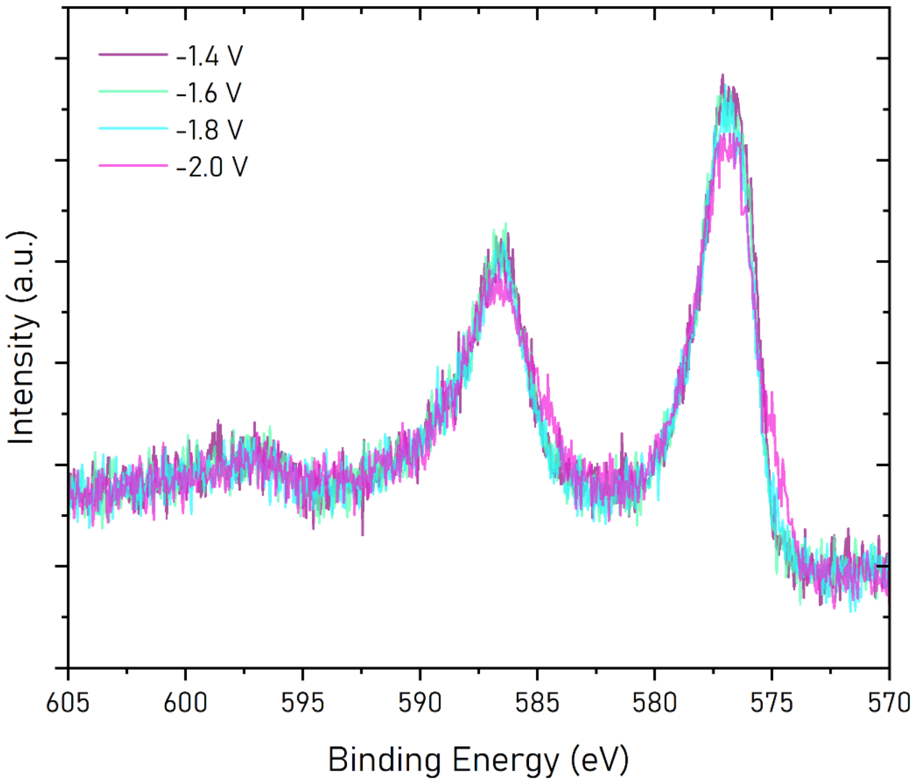

Supporting Figure 3: XPS measurements of Cr in the range of -1.4 to -2.0 V against 1 bar O_2.

Supporting Figure 3 shows EXACT-XPS measurements of the Cr in the range of -1.4 to -2.0 V. Within the, for the conclusions of the publication relevant, potential range from -1.4 V to -1.8 V vs. 1 bar O_2, no detectable change in the Cr oxidation state was observed. In contrast, the Mn spectra show a clear and quantifiable reduction from Mn^{3+} to Mn^{2+} over the same potential window (see Figure 7 of the Main Publication). This provides direct experimental evidence that Cr remains predominantly in the Cr^{3+} state during the main charge-storage process. At potentials below -1.8 V, small changes in the Cr spectra can be observed. However, these changes cannot be reliably quantified due to the lack of suitable reference spectra for Cr^{2+} in comparable chemical environments. We therefore explicitly refrain from assigning these features to a specific oxidation state. While these spectral changes may hint that the third low-potential capacitance peak could involve partial Cr reduction, the available data does not allow a definitive conclusion.

3. Experimental

For a better, real-life understanding of the architectures, photographs of both the half-cell and microelectrode samples have been taken and can be seen in Supporting Figure 4.

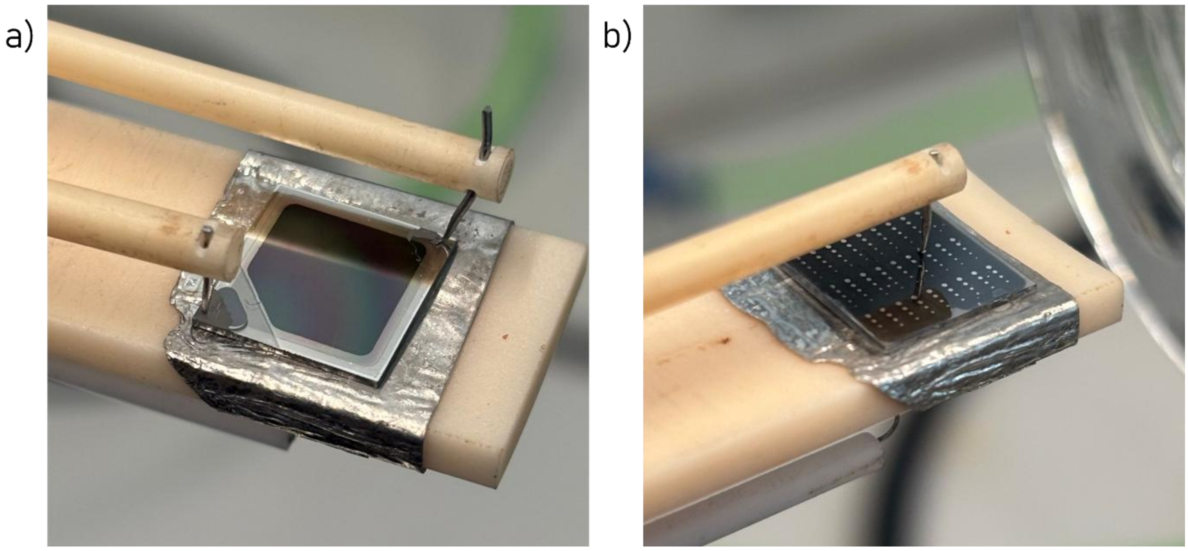

Supporting Figure 4: Photographs a) the half-cell and b) microelectrode samples.

[1] G. M. Rupp, A. Schmid, A. Nenning, and J. Fleig, Journal of The Electrochemical Society, 2016, 163 (6), F564-F573

[2] A. Nenning, S. Breitwieser, C. Melcher and J. Fleig, J. Mater. Chem. A, 2025,13, 29147-29160

[3] M. C. Biesinger, B. P. Payne, A. P. Grosvenor, L. W. Lau, A. R. Gerson and R. S. Smart, Applied Surface Science, 2011, 257, 2717–2730.

[4] E. S. Ilton, J. E. Post, P. J. Heaney, F. T. Ling and S. N. Kerisit, Applied Surface Science, 2016, 366, 475–485
